# Theoretical Study of the Photoisomerization Mechanism of All-*Trans*-Retinyl Acetate

**DOI:** 10.1021/acs.jpca.1c05533

**Published:** 2021-09-21

**Authors:** Michał Andrzej Kochman, Krzysztof Palczewski, Adam Kubas

**Affiliations:** † Institute of Physical Chemistry, Polish Academy of Sciences, Ul. Marcina Kasprzaka 44/52, 01-224 Warszawa, Poland; ‡ Department of Ophthalmology, Gavin Herbert Eye Institute, University of California, Irvine, California 92697, United States; § Department of Physiology and Biophysics, University of California, Irvine, California 92697, United States; ∥ Department of Chemistry, University of California, Irvine, California 92697, United States

## Abstract

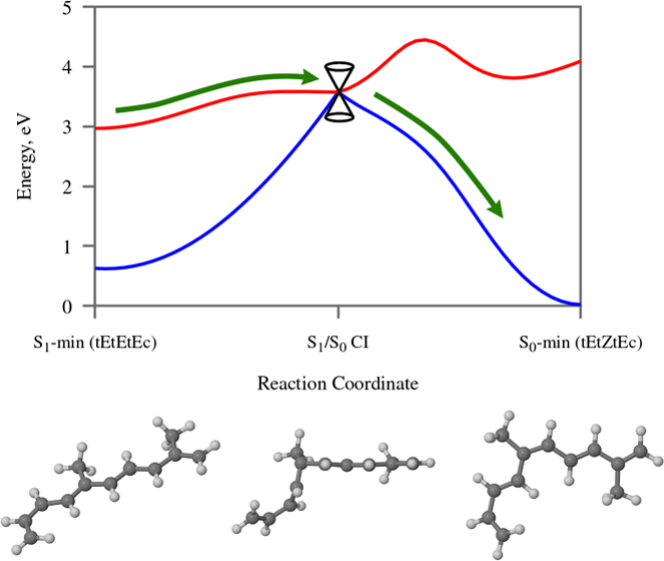

The compound 9-*cis*-retinyl acetate (9-*cis*-RAc) is a precursor to 9-*cis*-retinal, which has potential application in the treatment of some hereditary diseases of the retina. An attractive synthetic route to 9-*cis*-RAc is based on the photoisomerization reaction of the readily available all-*trans*-RAc. In the present study, we examine the mechanism of the photoisomerization reaction with the use of state-of-the-art electronic structure calculations for two polyenic model compounds: *tEtEt*-octatetraene and *tEtEtEc*-2,6-dimethyl-1,3,5,7,9-decapentaene. The occurrence of photoisomerization is attributed to a chain-kinking mechanism, whereby a series of S_1_/S_0_ conical intersections associated with kinking deformations at different positions along the polyenic chain mediate internal conversion to the S_0_ state, and subsequent isomerization around one of the double bonds. Two other possible photoisomerization mechanisms are taken into account, but they are rejected as incompatible with simulation results and/or the available spectroscopic data.

## 1 Introduction

Retinoids are a class of lipophilic compounds chemically related to vitamin A. In terms of structure, they consist of a polyenic chain with a polar functional group on one end (a hydroxyl group in retinol, an aldehyde group in retinal, etc.), and a six-membered β-ionone ring on the other end. They serve several biological functions, including acting as the chromophores of light-sensitive retinylidene proteins such as rhodopsin.^1,2^

In certain hereditary human diseases, such as Leber’s congenital amaurosis, mutations in genes encoding the proteins involved in the visual cycle disrupt the metabolism of retinoids, leading to vision impairment and loss.^3−7^ Beginning in the early 2000s, a specific retinoid, 9-*cis*-retinal, has been investigated as a therapeutic agent for the treatment of some of these diseases.^8−18^ (The designation “9-*cis*” and others like it refer to the location, in the polyenic chain, of a double bond with the cis configuration.) The pharmacological activity of 9-*cis*-retinal relies on the fact that it combines with opsin to form isorhodopsin,^13^ an analogue of rhodopsin which is also sensitive to light.^19,20^ Crucially, 9-*cis*-retinal is stable in the acidic environment of the stomach, so it can be administered orally, as opposed to intraocular injection.^6,10,18^

The potential therapeutic application of 9-*cis*-retinal creates the need for an efficient and scalable synthetic route to 9-*cis*-retinoids. Recently, Kahremany and co-workers^21^ proposed a synthetic strategy that uses as its starting point the readily available all-*trans*-retinoids. In that study, a set of 20 commercially available transition metal-based catalysts were screened for the conversion of all-*trans*-retinoids into mono-*cis* isomers. Encouragingly, the best-performing catalysts from among those considered in ref (21) achieved favorable regioselectivity, namely, a preference for the formation of the desired 9-*cis* isomers and moderately high conversion yields.

**Figure 1:**
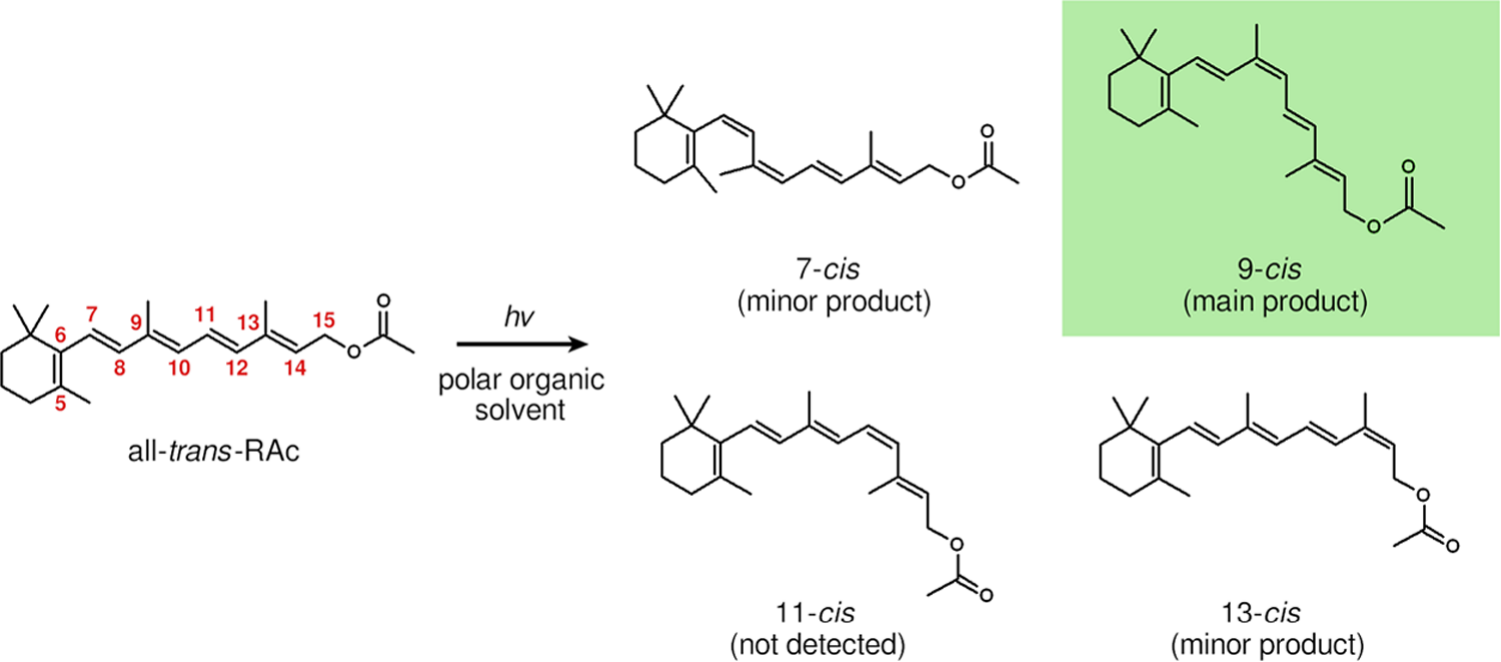
Photoisomerization reaction of all-*trans*-RAc. The main product is the 9-*cis* isomer. The 7-*cis* and 13-*cis* isomers appear as unwanted side products. The 11-*cis* isomer is not detected in the product mixture. Atom numbering is shown in red. Based on the findings presented in ref (39).

Under irradiation in the ultraviolet range, retinoids in organic solvents undergo cis ⇌ trans photoisomerization reactions.^22−38^ This process has long been employed as a synthetic route to mono-*cis* isomers, although its usefulness has been limited by its poor regioselectivity (the formation of multiple mono-*cis* isomers in comparable quantities). In a follow-up study, Kahremany et al.^39^ optimized the photoisomerization reaction for the synthesis of 9-*cis*-retinoids. To this end, a series of experiments were carried out with the aim of identifying the reaction conditions which would maximize the yield of 9-*cis* isomers. The conversion yield and product distribution ratio were found to depend on several factors, among them the choice of the all-*trans* retinoid substrate (retinol, retinal, retinoic acid, or retinyl acetate), the solvent in which the reaction was performed, and the irradiation wavelength. The highest yields of the 9-*cis* isomer were obtained with retinyl acetate (RAc) in polar organic solvents, such as acetonitrile or ethanol. This reaction setup, which is depicted schematically in Figure 1, was singled out as the most practical photochemical route to 9-*cis*-retinoids. As a proof of concept, it was used to synthesize gram-scale quantities of 9-*cis*-RAc.

The simplicity and catalyst-free nature of the photoisomerization reaction of all-*trans*-RAc makes it an attractive option for large-scale synthesis of 9-*cis*-retinoids. At the same time, however, the mechanism of photoisomerization is not well understood. The uncertainty extends to its basic aspects, such as the question of which excited electronic states are involved. A survey of the literature on the photoisomerization reactions of retinoids and other polyenic compounds suggests that several possible mechanisms must be considered, and we will now review each of these in turn. (For a more general overview of the photophysics of polyenes and related compounds, the reader is referred to refs (31, 32, 40−44)).

Chronologically, the first model of the photochemical reactivity of all-*trans*-RAc was constructed by Jayathirtha Rao and Bhalerao^34^ on the basis of the relationship between the reaction conditions and the product distribution (Figure 1). Within the framework of that first model, there are two reaction pathways for photoisomerization of all-*trans*-RAc. The first pathway is initiated by irradiation in the ultraviolet range and takes place in the manifold of singlet states. It is hypothesized that photoexcited all-*trans*-RAc converts into a zwitterionic intermediate in which the polyenic chain is twisted around the C9=10 double bond, and the C5=C6–C7=C8–C9 fragment donates charge onto the C10–C11=C12–C13=C14 fragment.^34^ The majority of this intermediate subsequently transforms into the 9-*cis* isomer.^34^ A small fraction instead undergoes decomposition by losing an acetate anion, which is followed by further reactions.^34^ It is assumed that the zwitterionic intermediate is stabilized by polar solvation, and this is taken as the explanation for the increased yields of isomerization and decomposition in polar solvents.^34^

Concerning the singlet pathway for photoisomerization, the involvement of a zwitterionic intermediate is difficult to reconcile with the fact that the fluorescence spectrum of all-*trans*-RAc does not show a signal which could be attributed to a highly polar species.^45−47^ Moreover, the electric dipole moment of the fluorescent state of all-*trans*-RAc was experimentally measured as 2.7 ± 0.2 D (debye),^47^ only marginally larger in magnitude than the electric dipole moment of the ground state, which is 2.33 D.^48^ Together, these considerations suggest that the intermediate, or intermediates, in the singlet pathway are not, in fact, zwitterionic, so the explanation for the solvent effect must lie elsewhere.

The second photoisomerization pathway comes into play when the reaction is carried out with the use of a sensitizer that promotes the population of the triplet states of all-*trans*-RAc.^34^ The mechanism of sensitization presumably involves triplet–triplet energy transfer from the sensitizer to all-*trans*-RAc.^26,33,49^ Under these conditions, all-*trans*-RAc undergoes isomerization into 9-*cis* and 13-*cis* isomers.^34^ The photostationary state consists of the all-*trans*, 9-*cis*, and 13-*cis* isomers in a ratio of roughly 7:1.5:1.5.^34^ The yield of the 9-*cis* isomer is too low for the sensitized reaction to be suitable for its synthesis.

An important point is that the intrinsic quantum yield of intersystem crossing (ISC) into the triplet manifold is only a few percent.^50^ This suggests that in the absence of a sensitizer, photoisomerization mainly takes place in the singlet manifold, hence leading to a different product distribution than in the sensitized reaction. For this reason, in what follows, we have narrowed our attention to photoisomerization in the singlet states.

Aside from the zwitterionic intermediate mechanism proposed in ref (34), two other scenarios can be envisioned for the photoisomerization of all-*trans*-RAc in the singlet manifold. The first is the chain-kinking mechanism, which was formulated in a series of theoretical studies by Olivucci, Robb, and co-workers.^51−55^ It is a general mechanism for the *E* ⇌ *Z* photoisomerization of polyenes, and it may therefore be applicable to all-*trans*-RAc. (As a side note on nomenclature, when referring to specific stereoisomers of polyenes, we use a notation in which the prefixes *E* and *Z* indicate the configurations of double bonds, while the prefixes *t* and *c* are used for single bonds.)

**Figure 2:**
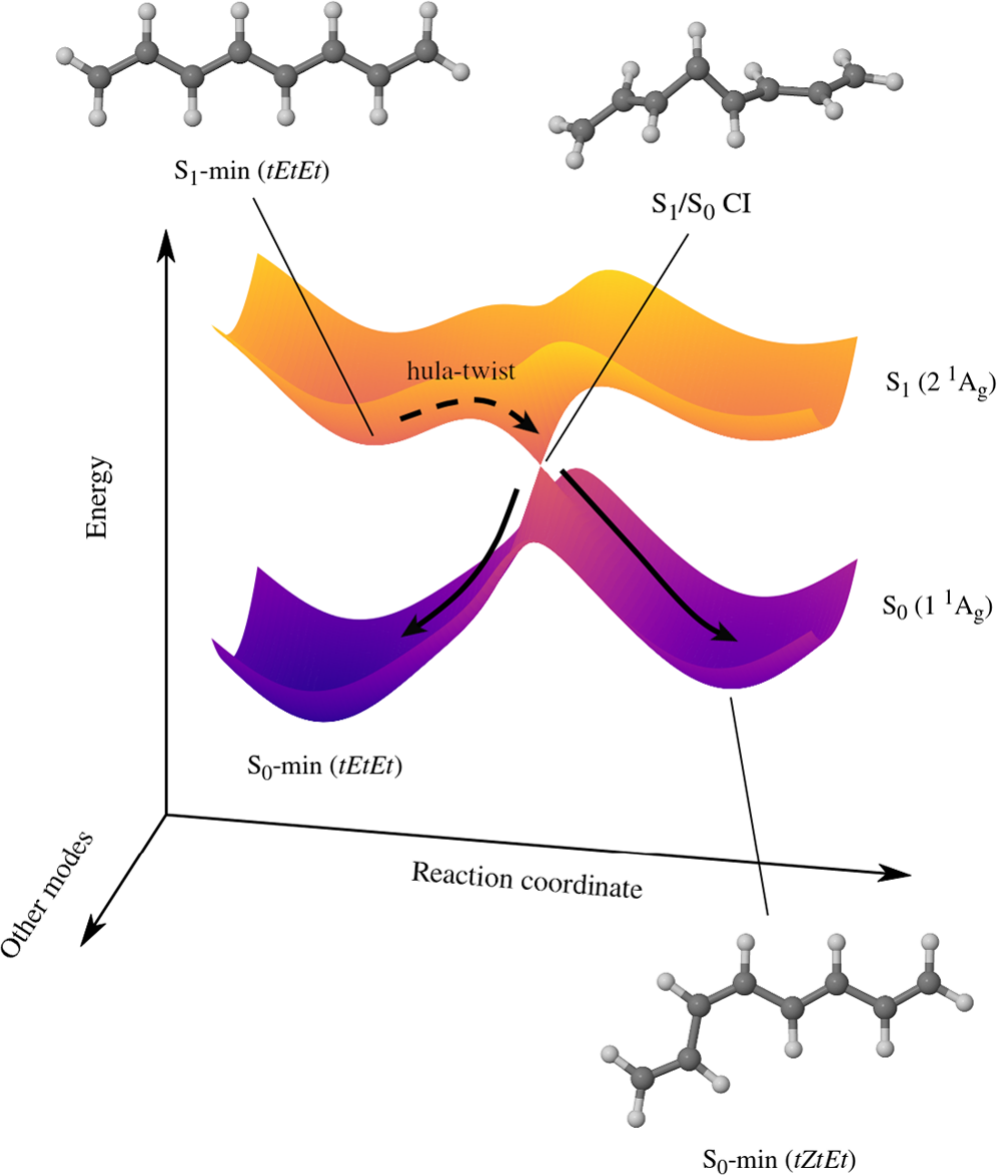
Overview of the chain-kinking mechanism^51−55^ on the example of *tEtEt*-OT. The reaction coordinate corresponds to torsions around C3=C4 and C4–C5 bonds. Based on the findings presented in ref (54).

Figure 2 shows the basic principles of the chain-kinking mechanism on the example of *tEtEt*-1,3,5,7-octatetraene (*tEtEt*-OT). In this mechanism, the starting point for isomerization is a minimum on the potential energy surface (PES) of the S_1_ (2 ^1^A_g_) state, which is labeled S_1_-min (*tEtEt*) in Figure 2. This minimum corresponds to a planar geometry of *C*_2*h*_ symmetry. Isomerization begins when the molecule undergoes rotation simultaneously around the C3=C4 double bond and the C4–C5 bond. The reaction path leads through a transition state on the PES of the S_1_ state, whose presence means that the photoisomerization of *tEtEt*-OT is an activated process. Further along the reaction path, the system encounters a conical intersection (CI) between the S_1_ and S_0_ states. At the CI structure, the polyenic chain exhibits a characteristic bend or kink-like deformation. The simultaneous rotation around the C3=C4 double bond and the C4–C5 bond, which takes the molecule from the S_1_-min (*tEtEt*) structure to the S_1_/S_0_-CI, is an instance of a hula-twist rotation^56−61^—a concerted rotation around a pair of consecutive double and single bonds. This type of intramolecular rotation has also been implicated in the photoisomerization processes of other chromophores such as protonated Schiff bases,^44,56−60,62−64^ the green fluorescent protein chromophore,^65−71^ and stilbene and its derivatives.^72−75^

At the S_1_/S_0_ CI, the molecule undergoes internal conversion from the S_1_ state to the S_0_ state. Once in the latter state, some fraction of the population relaxes toward the minimum which corresponds to the *tZtEt* isomer, while the remainder relaxes to the minimum of the *tEtEt* isomer. The relative yields of the *tZtEt* and *tEtEt* isomers are not known in quantitative terms, but extrapolating from the case of *tEt*-1,3,5-hexatriene, which has a low quantum yield of photoisomerization,^76^ the predominant outcome is expected to be relaxation to the *tEtEt* isomer.

**Figure 3:**
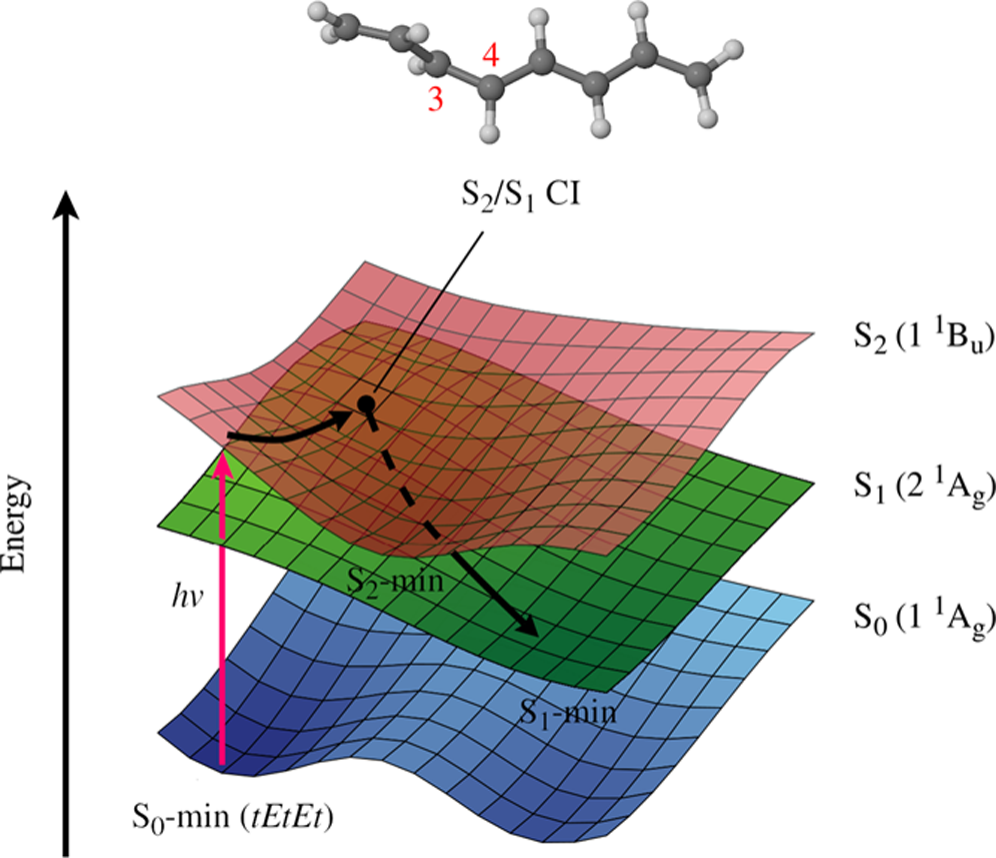
Mechanism of S_2_ → S_1_ internal conversion in *tEtEt*-OT according to Qu and Liu.^77^ The horizontal plane represents the motions of nuclei. Based on the findings presented in ref (77).

Qu and Liu^77^ performed nonadiabatic molecular dynamics simulations of the relaxation process of photoexcited *tEtEt*-OT and proposed a different mechanism for photoisomerization. As shown in Figure 3, the key feature of that mechanism is that photoisomerization is related to internal conversion from the initially excited S_2_ (1 ^1^B_u_) state to the S_1_ (2 ^1^A_g_) state.^77^ In linear polyenes, the two low-lying singlet ππ*-type excited states are 2 ^1^A_g_ and 1 ^1^B_u_ states.^78−80^ The 1 ^1^B_u_ state is dipole-allowed, and it is dominated by the singly excited HOMO^1^ LUMO^1^ configuration. On the other hand, the 2 ^1^A_g_ state is dipole-forbidden, and it has a large contribution from the doubly excited HOMO^0^ LUMO^2^ configuration.^81^ In the case of *tEtEt*-OT and longer linear polyenes, the 1 ^1^B_u_ state is located slightly above the 2 ^1^A_g_ state.^78,80^ As a consequence, shortly after photoexcitation, *tEtEt*-OT undergoes internal conversion from the initially excited S_2_ (1 ^1^B_u_) state to the S_1_ (2 ^1^A_g_) state.^82^

According to Qu and Liu, this internal conversion process takes place at a CI between S_2_ and S_1_ states, where the C3=C4 bond is twisted by roughly 107°.^77^ Afterward, some fraction of the excited-state population continues moving along the torsional coordinate and eventually reaches the *tZtEt* minimum on the S_1_ state.^77^ The remainder is reflected toward the *tEtEt* minimum.^77^ Because in this mechanism, the reaction coordinate involves twisting around one of the inner C=C bonds, we will refer to it as the one-bond-flip mechanism. The chain-kinking and the one-bond-flip mechanisms are not mutually exclusive: the former is concurrent with the S_2_ → S_1_ internal conversion of *tEtEt*-OT, while the latter kicks in at a later stage of the excited-state lifetime, when the molecule is already in the S_1_ state.

Unlike the chain-kinking mechanism, the one-bond-flip mechanism has not been explicitly generalized to polyenes other than OT. A case can be made that the one-bond-flip mechanism may potentially be operative in 1,3,5,7,9-decapentaene (DP) and longer linear polyenes, as these systems have the same energy ordering of low-lying excited states as *tEtEt*-OT, with the 1 ^1^B_u_ state being located slightly above the 2 ^1^A_g_ state.^78,80^ As a matter of fact, a somewhat similar photoisomerization mechanism was assumed in the original study of Kahremany et al.^39^ In this case, the ground- and excited-state PESs of all-*trans*-RAc and all-*trans*-retinal were mapped out along reaction paths for photoisomerization around double bonds in the polyenic chain. In these calculations, excited electronic states were described with the use of approximate methods rooted in density functional theory (DFT): time-dependent DFT (TD-DFT) and broken-symmetry DFT (DFT-BS).^83,84^ A crucial point is that both these approximate methods were able to treat the 1 ^1^B_u_ state, but they could not provide a realistic description of the 2 ^1^A_g_ state, due in part to it having a large contribution from doubly excited configurations.^81,85^ As a consequence, in these calculations, the 2 ^1^A_g_ state was either not detected or was not described correctly. Thus, Kahremany et al.^39^ did not locate a crossing between 1 ^1^B_u_ and 2 ^1^A_g_ states. Instead, rotation around a double bond was predicted to lead to a crossing of the 1 ^1^B_u_ state with the 1 ^1^A_g_ state. Despite the omission of the crossing with the 2 ^1^A_g_ state, the photoisomerization mechanism envisioned by Kahremany et al. does show some analogies to the one proposed by Qu and Liu. In both these mechanisms, the photoisomerization proceeds through a one-bond-flip motion, which occurs while the system is in the 1 ^1^B_u_ electronic state.

Importantly, the mechanism formulated in ref (39) showed some success in predicting the regioselectivity of the photoisomerization reaction. To explain the regioselectivity, a local polarization change (LPC) model was proposed, which relates the contributions of individual atoms to HOMO and LUMO orbitals with the ability of each double bond to “store” the electronic excitation energy. Within the framework of the LPC model, the C9–C10 bond was predicted to store an especially large amount of energy, which would imply efficient isomerization around that bond.

At the same time, however, there are also arguments against the involvement of the one-bond-flip mechanism in photoisomerization linear polyenes. Lyskov and co-workers^86^ simulated the excited-state relaxation process of *tEtEt*-OT using a substantially different methodology than was used in ref (77), namely, the multiconfigurational time-dependent Hartree^87,88^ (MCTDH) method with PESs parameterized at the combined density functional theory and multireference configuration interaction^89,90^ (DFT/MRCI) level. These authors did not observe photoisomerization during the S_2_ → S_1_ internal conversion process.^86^ Instead, the occurrence of photoisomerization was provisionally ascribed to the chain-kinking mechanism.^86^

Clearly, knowing the mechanism underlying the photoisomerization of all-*trans*-RAc is a prerequisite for understanding its regioselectivity, and the relationship between the reaction conditions and the product distribution. Therefore, the present study aims to identify the mechanism at work and to tie it in with the available experimental and computational data on the photophysics of all-*trans*-RAc. To this end, we have carried out high-level electronic structure calculations of optical properties and ground and excited-state PESs of polyenic models of that compound.

**Figure 4:**
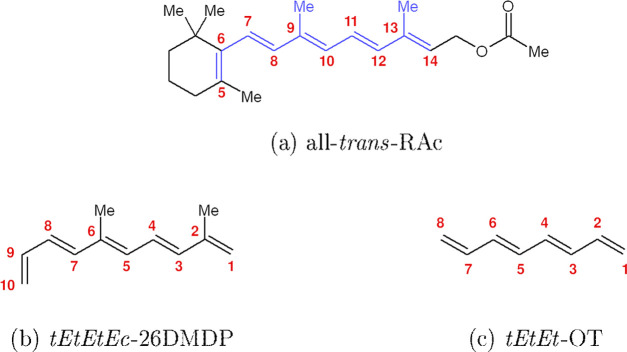
Comparison of the molecular structures of (a) all-*trans*-RAc, (b) its truncated model, *tEtEtEc*-26DMDP, and (c) *tEtEt*-OT. Atom numbering is shown in red. In panel (a), the fragment of the all-*trans*-RAc molecule that is retained in *tEtEtEc*-26DMDP is highlighted in blue.

In the first part of our study, we attempted to answer the question of whether photoisomerization can take place during the S_2_ → S_1_ internal conversion process, as per the one-bond-flip mechanism proposed in ref (77). For this purpose, we employed *tEtEt*-OT as a generic model of a polyenic chromophore. *tEtEt*-OT is also convenient in that it affords a direct comparison to the results presented in refs (54, 77, 86). The detailed discussion of these simulations is given in Section S2 of the Supporting Information. In the event, we have found that although the one-bond-flip mechanism may enter the picture for *tEtEt*-OT itself, it is unlikely to play a role in the photoisomerization of all-*trans*-RAc. Accordingly, we turned our attention to the chain-kinking mechanism. At this stage, we switched to *tEtEtEc*-2,6-dimethyl-1,3,5,7,9-decapentaene (*tEtEtEc*-26DMDP) as a more realistic computational model of all-*trans*-RAc. As illustrated in Figure 4, *tEtEtEc*-26DMDP includes the entire conjugated π-bonding system of all-*trans*-RAc. The acetate ester group was deleted from the model because it is electronically decoupled from the polyenic chain. The alkyl fragment of the β-ionone ring is photochemically inert, and it was likewise removed. On the other hand, the methyl groups at atoms C9 and C13 (which become atoms C6 and C2 in 26DMDP) were retained, as they may potentially affect the regioselectivity of photoisomerization.

## 2 Computational Methods

### 2.1 Electronic Structure Calculations

The electronic structures of model compounds were obtained with the use of extended multistate complete active space second-order perturbation theory^91^ (XMS-CASPT2), which is one of the most accurate methods available for molecules of this size.^92−95^ All calculations were performed in vacuum (that is to say, for isolated molecules); the investigation of the nature and effect of interactions with the solvent is relegated for future research.

The XMS-CASPT2 calculations were performed with the program BAGEL,^96,97^ version 1.1.2. For each of the compounds under study, the active space of the complete active space self-consistent field^98^ (CASSCF) reference calculation consisted of all those π- and π*-type orbitals which arise mainly from carbon 2p atomic orbitals. The choice of active space orbitals is shown in Figure S1 in the Supporting Information.

A certain complication arose regarding the choice of the state-averaging (SA) scheme in reference CASSCF calculations. As already noted by other authors,^78,99^ the energy ordering of the low-lying singlet excited states of linear polyenes changes on going from the CASSCF level of theory to correlated multireference methods. The change of energy ordering occurs because excitation energies into the various states of linear polyenes are unequally affected by the inclusion of dynamical electron correlation, a phenomenon known as differential correlation. In particular, the excitation energy into the dark 2 ^1^A_g_ state is rather insensitive to the inclusion of dynamical correlation, whereas the excitation energy into the bright 1 ^1^B_u_ state is very sensitive. The CASSCF method, which accounts for only a small amount of dynamical correlation, strongly overestimates the excitation energy into the 1 ^1^B_u_ state. The inclusion of dynamical electron correlation lowers the excitation energy into that state, bringing it into closer agreement with experiment.

To deal with the abovementioned deficiency of the CASSCF method, the number of states included in the state-averaging scheme in a given calculation was chosen according to circumstances. In situations where we were only interested in the 1 ^1^A_g_ and 2 ^1^A_g_ states of OT or 26DMDP, and not in any of the higher singlet states, we imposed state averaging over the two lowest singlet states with equal weights (SA-2-CASSCF). Likewise, two states were included at the stage of the XMS-CASPT2 calculation. In this case, the calculation only detects 1 ^1^A_g_ and 2 ^1^A_g_ states. We denote such two-state calculations with the acronym XMS(2)-CASPT2.

When, on the other hand, we are interested in the 1 ^1^B_u_ state, it is necessary to include in the state-averaging scheme a large enough number of states to allow the CASSCF calculation to detect that state. (The same number of states is then included in the subsequent XMS-CASPT2 calculation.) In preliminary test simulations, we found that for both *tEtEt*-OT and *tEtEtEc*-26DMDP, the 1 ^1^B_u_ state was detected, and CASSCF calculations were numerically reasonably stable, when six states were included with equal weights (SA-6-CASSCF). Therefore, in those calculations which aimed to characterize the 1 ^1^B_u_ state, we decided to include six states, a fact which we denote with the acronym XMS(6)-CASPT2. The test simulations did however also show that SA-6-CASSCF calculations are, on the whole, less stable than SA-2-CASSCF calculations. For this reason, we resorted to six-state calculations only when we were specifically interested in the 1 ^1^B_u_ state. Otherwise, we defaulted to two-state calculations.

Because the program BAGEL does not analyze, or take advantage of, molecular symmetry, the symmetries of electronic states were assigned manually. This was achieved by inspecting orbital symmetries, the leading configurations of CASSCF reference wave functions, and XMS-CASPT2 rotation matrices.

A vertical shift of 0.5 *E*_h_ (hartree) was imposed at all times. Moreover, the so-called single-state single-reference (SS-SR) contraction scheme^100^ was used. We employed the cc-pVDZ basis set^101^ in combination with the default density fitting basis set from the BAGEL library.

### 2.2 Exploration of Potential Energy Surfaces

A significant part of the present study involved mapping out ground- and excited-state potential energy surfaces (PESs) of model polyenes. Geometry optimizations were carried out by interfacing BAGEL to the computational chemistry software package Gaussian 16, revision A.03.^102^ In this setup, Gaussian acts as a “wrapper” around BAGEL and carries out the geometry optimization by calling BAGEL for the energy and gradient. The XMS-CASPT2 gradients were calculated analytically via the algorithm of Park and Shiozaki.^103^ As per the default settings in Gaussian 16, the geometries of minima on PESs were optimized with the use of the Berny algorithm in redundant internal coordinates.^104−111^ Each of the optimized geometries was verified to correspond to a minimum on the PES by calculating normal modes numerically.

Internal conversion is typically mediated by conical intersections (CIs) between the relevant electronic states. For this reason, we searched for minimum-energy conical intersection (MECI) geometries along the CI seams of the compounds under study. The MECI geometries were optimized using the penalty function method of Ciminelli et al.^112^ Within that method, the geometry optimization proceeds by minimizing the penalty function *f*(**R**) defined as
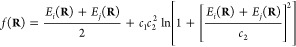
1where **R** denotes the molecular geometry and *E*_*i*_(**R**) and *E*_*j*_(**R**) are the energies of the intersecting states *i* and *j*. The role of the first term is to minimize the average of the energies of the intersecting states, while the second term is a restraint that ensures that the optimization procedure approaches and then remains on the CI seam. The parameter *c*_1_ controls the relative weights of first and second terms, and *c*_2_ controls the “rate” at which the optimization approaches the CI seam. We adopted the values recommended in ref (112): *c*_1_ = 5 (kcal/mol)^−1^ and *c*_2_ = 5 kcal/mol.

As with the optimizations of minima on PESs, the optimizations of MECI geometries were performed by interfacing BAGEL to Gaussian 16, and taking advantage of the Berny algorithm, which is implemented in the latter program. This is made possible by the fact that the optimization of a minimum of the penalty function *f*(**R**) is entirely analogous to the optimization of a minimum on a PES. In the course of MECI optimizations, the value and the gradient of *f*(**R**) were calculated from the energies of intersecting PESs (*E*_*i*_(**R**) and *E*_*j*_(**R**) ) and their analytical gradients and were passed on to Gaussian 16.

Moreover, we performed a set of PES scans that examined the ground- and excited-state PESs of OT and 26DMDP. More specifically, we mapped out the topography of the S_2_/S_1_ CI seam of *tEtEt*-OT, and we scanned the PESs of the S_1_ and S_0_ states of 26DMDP along reaction paths for *E* → Z photoisomerization. The technical details of these PES scans are given in Section 3.3 and in Section S2.2 of the Supporting Information.

## 3 Results and Discussion

### 3.1 Excited Electronic States of 2,6-Dimethyl-1,3,5,7,9-decapentaene (26DMDP)

2,6-Dimethyl-1,3,5,7,9-decapentaene (26DMDP) is our model compound for investigating the regioselectivity and product distribution of the photoisomerization reaction of all-*trans*-RAc. Our first order of business will be to examine its excited electronic states. The vertical excitation spectrum of 26DMDP in its *tEtEtEc* isomeric form is summarized in Table 1. Although the ground-state equilibrium geometry is slightly nonplanar and does not possess any symmetry elements other than the identity relation, the analysis of its excited states is simplified using the symmetry labels of the *C*_2*h*_ point group, which is the point group of unsubstituted linear polyenes. For this reason, we classified the excited states of *tEtEtEc*-26DMDP according to their symmetry in the *C*_2*h*_ point group.

**Table 1:** Vertical Excitation Spectrum of *tEtEtEc*-26DMDP—Vertical Excitation Energies (Δ*E*) and Associated Oscillator Strengths (*f*)^a^

state	Δ*E*, eV	*f*	μ, debye
S_0_ (1 ^1^A_g_)			0.60
S_1_ (2 ^1^A_g_/1 ^1^B_u_)	4.041	0.714	0.49
S_2_ (2 ^1^A_g_/1 ^1^B_u_)	4.114	0.547	0.56
S_3_ (2 ^1^B_u_)	5.025	0.004	0.65
S_4_ (3 ^1^A_g_)	5.818	5 × 10^–4^	0.76
S_5_ (3 ^1^B_u_)	6.259	4 × 10^–4^	0.65

a μ is the magnitude of the (unrelaxed) electric dipole moment of the given state. The calculation was performed at the XMS(6)-CASPT2/cc-pVDZ level of theory at the ground-state equilibrium geometry optimized at the XMS(2)-CASPT2/cc-pVDZ level (see Figure 6a).

The lowest two singlet excited states (S_1_ and S_2_) of *tEtEtEc*-26DMDP are narrowly separated, with one being located at 4.041 eV and the other at 4.114 eV. Both exhibit appreciably large oscillator strengths for excitation from the S_0_ state. The S_1_ and S_2_ states can be interpreted as arising from mixing between a spectroscopically dark 2 ^1^A_g_-like state and a bright 1 ^1^B_u_-like state. The occurrence of mixing between these states is made possible by the fact that the ground-state equilibrium geometry of *tEtEtEc*-26DMDP deviates quite strongly from ideal *C*_2h_ symmetry.

Mixing between 2 ^1^A_g_ and 1 ^1^B_u_ states has a strong influence on the relaxation dynamics of retinylidene protonated Schiff base chromophores.^113,114^ The possibility presents itself that this effect may also play a role in the photoisomerization mechanism of all-*trans*-RAc, for example, by altering the topography of excited-state PESs. Mixing between these states also has a bearing on the setup of XMS-CASPT2 calculations for 26DMDP because it can only be detected when a large enough number of states is included. To investigate this effect, we optimized the minimum on PES of the S_1_ state of *tEtEtEc*-26DMDP with an XMS(6)-CASPT2/cc-pVDZ treatment of electronic structure. Having done so, we found that the S_1_ state at the minimum has a 2 ^1^A_g_-like character. The oscillator strength for S_1_ → S_0_ fluorescence emission takes a very low value of 4 × 10^–4^, indicating that the S_1_ state at the minimum is decidedly dark and 2 ^1^A_g_-like, with no significant “admixture” of the bright 1 ^1^B_u_ state. In other words, the mixing between 2 ^1^A_g_ and 1 ^1^B_u_ states is eliminated by the relaxation of the molecule to the minimum on the S_1_ state. Hence, we expect that the state mixing will not affect the topographies of the PESs of S_1_ and S_2_ states in a significant way. For reference, the minimum on the S_1_ state of *tEtEtEc*-26DMDP optimized at the XMS(6)-CASPT2/cc-pVDZ level is shown in Figure S5 in the Supporting Information.

There is a large energy gap between the S_3_ state, and the narrowly spaced S_1_ and S_2_ states. It is clear that S_1_ and S_2_ states are the only states that can be populated by the irradiation of the lowest photoabsorption band of all-*trans*-RAc. The S_3_ state and all higher excited states are not expected to be involved in the photophysics of all-*trans*-RAc under these conditions.

Further on the subject of the vertical excitation spectrum of *tEtEtEc*-26DMDP, Table 1 also lists magnitudes of the unrelaxed electric dipole moment of each state. It can be seen that the singlet ground state and all five excited states obtained in the XMS(6)-CASPT2/cc-pVDZ calculation are essentially nonpolar, with very small electric dipole moments on the order of 1 D. To put that result into context, typical intramolecular charge-transfer excited states of organic molecules of comparable size exhibit dipole moments of some 5–10 D.^115−117^ Given the lack of a charge-transfer state among the low-lying excited states of *tEtEtEc*-26DMDP, it seems unlikely that the photoisomerization reaction of all-*trans*-RAc could be mediated by such a state.

### 3.2 Molecular Geometries of 26DMDP

By now, we have significantly narrowed down the possibilities regarding the photoisomerization mechanism of all-*trans*-RAc. To recapitulate, the hypothesis that photoisomerization begins during the S_2_ (1 ^1^B_u_) → S_1_ (2 ^1^A_g_) internal conversion process was rejected on the grounds that the topography of the S_2_/S_1_ CI seam favors internal conversion at near-planar geometries (see Section S2 of the Supporting Information). Moreover, we considered the possibility that all-*trans*-RAc undergoes photoisomerization while in an intramolecular charge-transfer state, but this seems unlikely in light of the fact our calculations for *tEtEtEc*-26DMDP have not revealed the existence of such a state. This leaves the chain-kinking mechanism,^51−55^ which begins in the S_1_ (2 ^1^A_g_) state, as the most plausible scenario for the photoisomerization reaction. We will now examine the functioning of that mechanism in the case of 26DMDP. In the present section, the focus is on the key molecular geometries: minima on the PESs of S_0_ and S_1_ states, and CIs between these states. The reaction paths for photoisomerization will be discussed in the next section.

On the technical side, the ground- and excited-state PESs of 26DMDP were treated at the XMS(2)-CASPT2/cc-pVDZ level. As noted in the previous section, at the Franck-Condon geometry (i.e., the ground-state equilibrium geometry) of *tEtEtEc*-26DMDP, there is mixing between the 2 ^1^A_g_ and the 1 ^1^B_u_ state, but the mixing vanishes at the geometry on the PES of the S_1_ state. A two-state XMS-CASPT2 calculation is therefore sufficient for the description of the PESs of S_1_ and S_0_ states.

**Figure 5:**
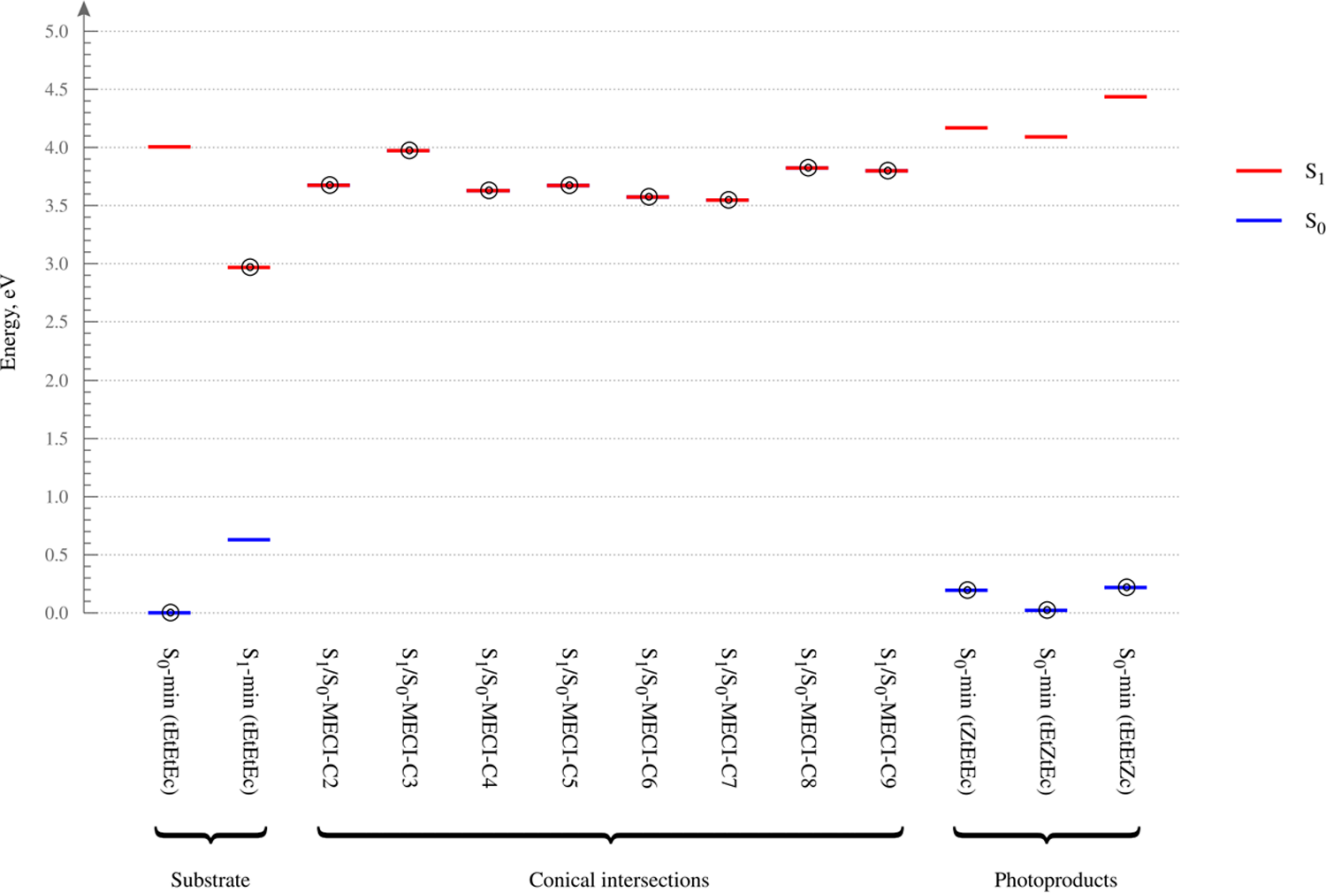
Energy level diagram for 26DMDP obtained at the XMS(2)-CASPT2/cc-pVDZ level of theory. The state (or, states, in the case of a MECI geometry) on which a given structure was optimized is marked with a bullseye symbol. The origin of the energy scale corresponds to the energy of the S_0_-min (*tEtEtEc*) structure. The relative energies do not include zero-point vibrational energy corrections, as these are not defined for a MECI geometry.

**Figure 6:**
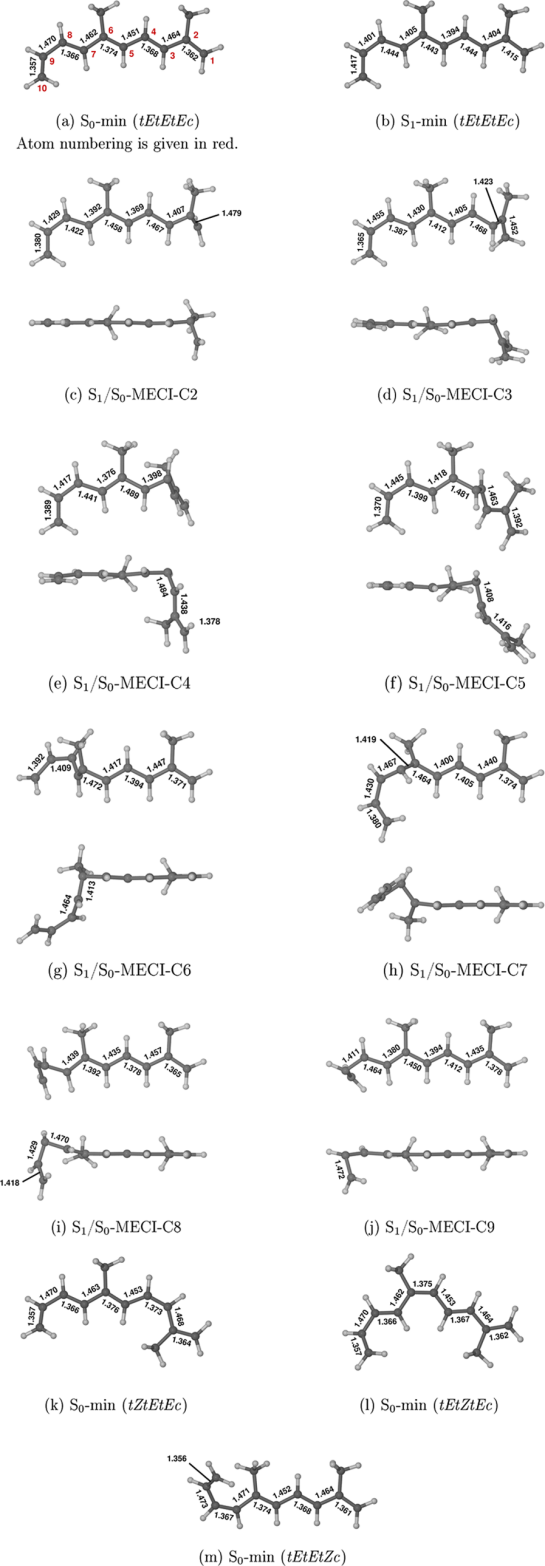
Geometries of 26DMDP as optimized at the XMS(2)-CASPT2/cc-pVDZ level of theory. Because MECI geometries feature strong deformations of the polyenic chain, for each of these structures, we show two views of the molecular geometry from different viewpoints. Selected bond lengths are given in units of ångström.

Because there are a large number of molecular structures to be considered, we present first the calculated energy level diagram for 26DMDP (Figure 5), which will serve as a kind of visual catalog. The various structures are also characterized in Table 2, and their geometries are shown in Figure 6. We will now examine each of these structures in turn.

**Table 2:** Characterization of the Relevant Ground- and Excited-State Geometries of 26DMDP—Energies of S_0_ and S_1_ States and Values of Torsion Angles within the Polyenic Chain^a^

structure	*E*(S_0_), eV	*E*(S_1_), eV	τ_1-2-3-4_, °	τ_2-3-4-5_, °	τ_3-4-5-6_, °	τ_4-5-6-7_, °	τ_5-6-7-8_, °	τ_6-7-8-9_, °	τ_7-8-9-10_, °
S_0_-min (*tEtEtEc*)	0	4.005	180.0	180.0	180.0	179.8	179.4	178.7	–27.6
S_1_-min (*tEtEtEc*)	0.629	2.967	180.0	180.0	180.0	180.0	180.0	180.0	0.0
S_1_/S_0_-MECI-C2	3.674	3.674	123.4	173.1	–178.4	–176.9	179.1	179.2	–0.5
S_1_/S_0_-MECI-C3	3.972	3.972	–117.1	110.1	178.5	–170.8	176.3	179.1	–6.8
S_1_/S_0_-MECI-C4	3.628	3.628	173.4	95.8	–128.6	–177.2	175.0	171.1	–2.3
S_1_/S_0_-MECI-C5	3.672	3.672	173.5	–135.8	–124.4	96.3	166.1	–172.8	2.9
S_1_/S_0_-MECI-C6	3.573	3.573	179.8	–173.9	177.0	105.6	–123.9	–124.0	–2.3
S_1_/S_0_-MECI-C7	3.546	3.546	179.1	–177.3	–172.7	118.4	115.6	–106.4	–4.4
S_1_/S_0_-MECI-C8	3.823	3.823	–179.9	179.6	–179.3	–167.1	161.2	90.6	44.6
S_1_/S_0_-MECI-C9	3.798	3.798	180.0	179.8	179.6	–178.3	175.2	171.9	–59.9
S_0_-min (*tZtEtEc*)	0.194	4.168	180.0	0.0	180.0	179.9	179.3	178.7	–27.6
S_0_-min (*tEtZtEc*)	0.022	4.091	180.0	–179.9	–179.8	0.3	–179.3	–178.6	28.5
S_0_-min (*tEtEtZc*)	0.218	4.436	180.0	–179.6	179.7	–179.7	160.6	–10.5	–44.6

a The energies are quoted relative to the energy of the S_0_ state at the ground-state equilibrium geometry of the *tEtEtEc* isomer. They do not include zero-point vibrational energy corrections, as these are not defined for a MECI structure. τ_1-2-3-4_, τ_2-3-4-5_, etc., are the successive torsion angles along the polyenic chain.

The ground-state equilibrium geometry of *tEtEtEc*-26DMDP, denoted S_0_-min (*tEtEtEc*), is shown in Figure 6a. (This is the same geometry as was used in the calculation of the vertical excitation spectrum of *tEtEtEc*-26DMDP in Section 3.1) Atoms C1–C9 and the carbon atoms of two methyl groups are roughly coplanar, while atom C10 is slightly displaced away from the molecular plane. This displacement is presumably caused by the steric interaction between the hydrogen atom of atom C7 and the nearer of the two hydrogen atoms of atom C10. As expected, the conjugated π-bonding system shows a pronounced bond length alternation, with short double bonds (C1=C2, C3=C4, C5=C6, C7=C8, and C9=10) being alternated by longer single bonds (C2–C3, C4–C5, C6–C7, and C8–C9).

We have located a single minimum on the PES of the S_1_ (2 ^1^A_g_) state, and its structure, denoted S_1_-min (*tEtEtEc*), is shown in Figure 6b. The existence of a minimum on the S_1_ state of *tEtEtEc*-26DMDP is in line with the fact that all-*trans*-RAc in organic solvents exhibits low but detectable quantum yields of fluorescence.^50,118^ The molecular geometry at the minimum is planar and belongs to the *C*_s_ point group. Moreover, there is an inversion of bond length alternation with respect to the ground-state equilibrium geometry: C1=C2, C3=C4, C5=C6, C7=C8, and C9=10 are now markedly elongated, while C2–C3, C4–C5, C6–C7, and C8–C9 bonds have contracted. This effect is characteristic of the 2 ^1^A_g_-type excited states of polyenes;^54,55,99^ in particular, it also occurs in *tEtEt*-OT (see Figure S3 in the Supporting Information).

*tEtEtEc*-26DMDP exhibits a series of MECI structures along the CI seam between S_0_ and S_1_ states, each of which is associated with a kinking deformation at one of the inner carbon atoms of the polyenic chain, namely, C2–C9. These are labeled S_1_/S_0_-MECI-C2 to S_1_/S_0_-MECI-C9, according to the carbon atom where the kinking occurs, and their geometries are shown in panels c–j of Figure 6. In all cases, the polyenic chain shows a well-developed kink—a marked twisting around one, two, or three consecutive C–C and C=C bonds. (A list of all relevant torsion angles in all optimized geometries is given in Table 2.) For example, in S_1_/S_0_-MECI-C6, there is twisting around C4–C5, C5=C6, and C6–C7 bonds. Hence, a fraction of molecules reaching S_1_/S_0_-MECI-C6 are expected to undergo *E* → *Z* isomerization around the C5=C6 bond, which is already strongly twisted at the MECI geometry. The C5=C6 bond of 26DMDP is a counterpart of the C9=10 bond of RAc, so this is analogous to the photoisomerization of all-*trans*-RAc into the 9-*cis* isomer.

S_1_/S_0_-MECI-C9 is a special case because it features a twist of the =C10H_2_ methylidene group around the axis of the C9=C10 bond. The C9=C10 bond of 26DMDP is the counterpart of the C5=C6 bond of RAc, which forms part of the β-ionone ring, and cannot undergo *E*/*Z* isomerization. Hence, S_1_/S_0_-MECI-C9 does not act as a gateway to double-bond photoisomerization. However, it may still be accessible because the β-ionone ring is somewhat flexible, so that internal conversion near S_1_/S_0_-MECI-C9 may contribute to the nonradiative deactivation process of all-*trans*-RAc.

The energies of the S_1_/S_0_-MECI structures of *tEtEtEc*-26DMDP fall in the range of roughly 3.5–4.0 eV relative to the energy of the S_0_ state at the ground-state equilibrium geometry. This energy range is substantially higher than the minimum on the S_1_ state, which is located at an energy of 2.967 eV. On the other hand, in the synthesis developed by Kahremany et al.,^39^ a solution of all-*trans*-RAc is irradiated at a wavelength of 385 nm, which corresponds to a photon energy of 3.22 eV. Thus, the amount of energy supplied to the molecule is actually slightly lower than the energies of S_1_/S_0_-MECI structures according to our calculations for *tEtEtEc*-26DMDP. It follows that internal conversion at S_1_/S_0_ CIs is an activated process.

We hypothesize that in all-*trans*-RAc, internal conversion predominantly takes place at a subset of the eight kinked S_1_/S_0_ CIs. Because each of the S_1_/S_0_ CIs (except S_1_/S_0_-MECI-C9) can act as a gateway for photoisomerization around one of the C=C double bonds, a preference for internal conversion at some of the S_1_/S_0_ CIs would provide a mechanistic basis for regioselectivity in the photoisomerization reaction. However, on the basis of our calculations alone, we cannot conclusively predict which CIs of all-*trans*-RAc (or, of *tEtEtEc*-26DMDP) are the most accessible.

Presumably, one of the factors that control the rate of internal conversion at a given S_1_/S_0_ CI is its energetic accessibility, i.e., its energy relative to the minimum on the S_1_ state (S_0_-min (*tEtEtEc*)). By our estimate, the XMS(2)-CASPT2/cc-pVDZ calculation can be expected to exhibit errors of up to around 0.1 eV for the relative energies of different locations on the PES of the S_1_ state. (We estimate that the uncertainty in the relative energies is the same as the error in the 0–0 excitation energy into the S_1_ (2 ^1^A_g_) state of *tEtEt*-OT calculated at the XMS(6)-CASPT2/cc-pVDZ level, which is 0.119 eV.) This is comparable in magnitude to the calculated energy differences between the eight S_1_/S_0_-MECI structures of *tEtEtEc*-26DMDP. We can be reasonably confident that S_1_/S_0_-MECI-C3, which has the highest energy from among the eight S_1_/S_0_-MECI structures of *tEtEtEc*-26DMDP, is inaccessible. We also know that S_1_/S_0_-MECI-C9 does not mediate double-bond photoisomerization, so the question of whether it is accessible is irrelevant to the regioselectivity of photoisomerization. However, that still leaves us with six S_1_/S_0_-MECI structures, all of which can potentially play a role in the photoisomerization mechanism.

The best way forward seems to be to attempt to correlate the simulation results with the composition of the photoproduct mixture in the synthesis developed by Kahremany et al.^39^ The main photoproduct under all sets of experimental conditions is 9-*cis*-RAc. Its formation corresponds to *E* → *Z* isomerization around the C5=C6 bond of *tEtEtEc*-26DMDP, leading to *tEtZtEc*-26DMDP. The molecular structure of the latter isomer is shown in Figure 6l. We ascribe its formation to internal conversion in the vicinity of S_1_/S_0_-MECI-C5, S_1_/S_0_-MECI-C6, and/or S_1_/S_0_-MECI-C7, as all of these structures feature twisting around the C5=C6 bond. Unfortunately, we cannot be certain which of these three structures makes the greatest contribution to isomerization around the C5=C6 bond, as the calculated energy differences among them are roughly the same as the estimated accuracy of our calculations. In any case, the fact that as many as three low-energy S_1_/S_0_-CIs of *tEtEtEc*-26DMDP can mediate isomerization around the C5=C6 bond provides a partial explanation for the regioselectivity of the photoisomerization reaction of all-*trans*-RAc. As will be discussed below, photoisomerizations around the other double bonds are more discriminating, with only one or two “gateway” S_1_/S_0_-CIs. This suggests that photoisomerization around the C5=C6 bond of *tEtEtEc*-26DMDP (which corresponds to the formation of 9-*cis*-Rac) is favored on probabilistic grounds.

13-*cis*-RAc appears as a minor side product under all sets of experimental conditions.^39^ Its formation corresponds to a rotation of the terminal =C1H_2_ methylidene group of *tEtEtEc*-26DMDP. The only S_1_/S_0_-MECI structure in which there is twisting around the terminal C1=C2 bond is S_1_/S_0_-MECI-C2, and internal conversion in the vicinity of that structure could conceivably lead to the formation of the 13-*cis* isomer.

Another minor side product, detected only in polar solvents, is 7-*cis*-RAc.^39^ Within the framework of our truncated model, the counterpart of 7-*cis*-RAc is *tEtEtZc*-26DMDP, in which it is the C7=C8 bond that has the *Z* geometry. For reference, the ground-state equilibrium geometry of *tEtEtZc*-26DMDP is shown in Figure 6m. The formation of *tEtEtZc*-26DMDP is attributable to internal conversion near S_1_/S_0_-MECI-C6, S_1_/S_0_-MECI-C7, and/or S_1_/S_0_-MECI-C8, as all three MECI structures feature twisting around the C7=C8 bond. According to our calculations, S_1_/S_0_-MECI-C6 and S_1_/S_0_-MECI-C7 are the lowest in energy from among the eight S_1_/S_0_-MECI structures of *tEtEtEc*-26DMDP, while S_1_/S_0_-MECI-C8 lies substantially higher in energy. Therefore, we attribute the formation of 7-*cis*-RAc to internal conversion at S_1_/S_0_-MECI-C6 and S_1_/S_0_-MECI-C7.

The one mono-*cis* isomer of RAc that was not detected in the photoproduct mixture under any experimental conditions is the 11-*cis* isomer.^39^ Its counterpart among the *E*/*Z* isomers of 26DMDP is *tZtEtEc*-26DMDP, in which the C3=C4 bond adopts a *Z* geometry (see Figure 6k). 26DMDP has two S_1_/S_0_-MECI structures (S_1_/S_0_-MECI-C3 and S_1_/S_0_-MECI-C4) which feature twisting around the C3=C4 bond and which could potentially act as a gateway for the formation of isomerization around that bond. (S_1_/S_0_-MECI-C5 features only a slight twisting around the C3=C4 and is unlikely to mediate photoisomerization around that bond.) According to our calculations, S_1_/S_0_-MECI-C3 lies relatively high in energy and can be discounted as inaccessible. On the other hand, S_1_/S_0_-MECI-C4 is low enough in energy that it may be accessible, and it may mediate photoisomerization around the C3=C4 bond of 26DMDP. This finding raises the possibility that the photoisomerization reaction of all-*trans*-RAc does, in fact, produce small amounts of 11-*cis* isomer as a minor product, and that this particular isomer went undetected in ref (39). Still, even if that were the case, from a practical standpoint, the formation of the 11-*cis* isomer does not seem to be a serious problem because that isomer can be cleanly separated from the desired 9-*cis* isomer with the use of a suitable HPLC procedure.^119^

### 3.3 Reaction Paths

This section extends the analysis of the photoisomerization mechanism by discussing reaction paths leading from the substrate (*tEtEtEc*-26DMP) to some of the photoproducts. For the sake of brevity, we focus on two representative reaction paths, of which one leads to *tEtZtEc*-26DMP (the counterpart of 9-*cis*-RAc) and the other to *tEtEtZc*-26DMP (the counterpart of 7-*cis*-RAc).

We generated the reaction paths using linear interpolations in internal coordinates (LIIC). The starting point of each reaction path was the S_1_-min (*tEtEtEc*) structure, the midpoint was one of the S_1_/S_0_-MECI structures, and the end point was the ground-state equilibrium geometry of the photoproduct isomer. The geometry of each of these structures was described with a system of internal coordinates. Afterward, the reaction path was generated via a linear interpolation between these structures in terms of the internal coordinates. The energies of the S_1_ and S_0_ states of 26DMDP were scanned along the resulting reaction paths by performing single-point calculations at the XMS(2)-CASPT2/cc-pVDZ level of theory. The interpolated geometries located along the reaction path were not reoptimized during the PES scan; rather, they were taken as is from the linear interpolation. As such, these PES scans were performed as unrelaxed scans.

**Figure 7:**
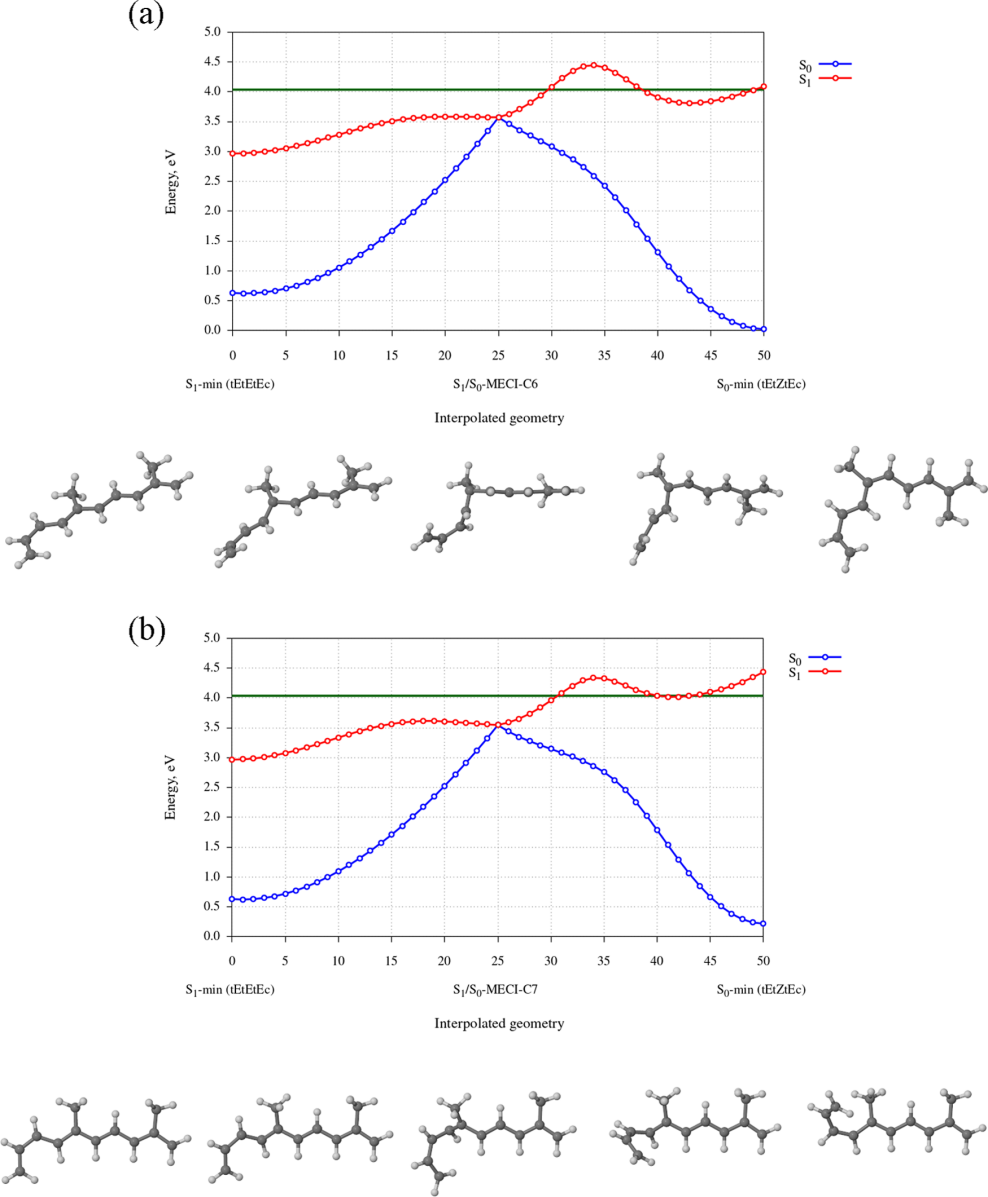
(a) Energies of the S_0_ and S_1_ states of 26DMDP along a reaction path leading from the S_1_-min (*tEtEtEc*), through S_1_/S_0_-MECI-C6, and to S_0_-min (*tEtZtEc*). The reaction path was generated through a LIIC procedure. The evolution of molecular geometry is shown in the insets at the bottom. The zero of the energy scale corresponds to the energy of the S_0_-min (*tEtEtEc*) structure. The horizontal green line indicates the amount of energy imparted on the molecule by an S_0_ → S_1_ vertical excitation, which is 4.041 eV according to the XMS(6)-CASPT2/cc-pVDZ calculation. (b) Same as in panel (a), but here the reaction path leads from S_1_-min (*tEtEtEc*), through S_1_/S_0_-MECI-C7, and to S_0_-min (*tEtEtZc*).

Figure 7a shows a scan of the energies of S_0_ and S_1_ states along a reaction path leading from S_0_-min (*tEtEtEc*), through S_1_/S_0_-MECI-C6, and finally to S_0_–min (*tEtZtEc*). Here, the *tEtEtEc*-26DMP structure is the 0th point along the reaction path, S_1_/S_0_-MECI-C6 is the 25th point, and S_0_–min (*tEtZtEc*) is the 50th point. Starting from the *tEtEtEc*-26DMP structure, S_1_/S_0_-MECI-C6 is reached through a simultaneous rotation around C5=C6, C6–C7, and C7=C8 bonds. This deformation of the polyenic chain can be described as a kinking deformation, or alternatively as a hula-twist rotation, with the distinction that in this case, three consecutive bonds are involved. As the molecule approaches the MECI geometry, the energy of the S_1_ state rises at first and then levels off. Meanwhile, the energy of the S_0_ state rises rapidly up until the S_1_/S_0_-MECI-C6 structure.

Importantly, it can be seen that the S_1_/S_0_-MECI-C6 structure lies low enough in energy that it can be accessed following an initial photoexcitation of the *tEtEtEc*-26DMDP molecule into the S_1_ state. As specified in Table 2, the S_1_/S_0_-MECI-C6 structure is located at an energy of 3.573 eV relative to the ground-state equilibrium geometry. On the other hand, the XMS(6)-CASPT2/cc-pVDZ calculation (see Table 1 in Section 3.1) predicts that an S_0_ → S_1_ vertical excitation imparts 4.014 eV on a molecule of the *tEtEtEc* isomer. This amount of energy is sufficient for the photoexcited molecule to reach the S_1_/S_0_-MECI-C6 structure while evolving on the PES of the S_1_ state.

At S_1_/S_0_-MECI-C6, the direction of the reaction path changes: rotation around the C5=C6 bond continues in the same direction as before, but rotation around the C6–C7 and C7=C8 bonds reverses direction. The reason for the change of direction is that in the course of photoisomerization, the configuration of the C5=C6 bond changes from *E* to *Z*, but C6–C7 and C7=C8 bonds are twisted only temporarily, and afterward, they revert to a *trans* configuration. The change of direction manifests itself as a sudden change in the slopes of the curves representing the energies of S_0_ and S_1_ states. As the molecule moves from S_1_/S_0_-MECI-C6 toward S_0_–min (*tEtZtEc*), the energy of the S_0_ state falls sharply. The fact that relaxation from S_1_/S_0_-MECI-C6 to S_0_–min (*tEtZtEc*) proceeds downhill in energy on the PES of the S_0_ state confirms that S_1_/S_0_-MECI-C6 acts as a gateway for isomerization around the C5=C6 bond. The steep slope of the PES of the S_0_ state in the direction toward S_0_–min (*tEtZtEc*) ensures that a fraction of the excited-state population that undergoes S_1_ → S_0_ internal conversion near S_1_/S_0_-MECI-C6 will subsequently relax to the S_0_–min (*tEtZtEc*) structure. As a side note, relaxation to S_0_–min (*tEtZtEc*) is strongly exhothermic, and the photoproduct will be formed in a vibrationally highly excited ground electronic state (a “hot” ground state). Double-bond isomerization may therefore be accompanied by rotamerizations around C–C single bonds within the polyenic chain.

Let us now move on to the reaction path for the formation of *tEtEtZc*-26DMP. Figure 7b shows a scan of the energies of S_1_ and S_0_ states along a reaction path starting at S_0_-min (*tEtEtEc*), leading through S_1_/S_0_-MECI-C7, and terminating at S_0_-min (*tEtEtZc*). It can be seen that this reaction path is similar to the one for isomerization around the C5=C6 bond. The first segment of the reaction path (from S_0_-min (*tEtEtEc*) to S_1_/S_0_-MECI-C7) again consists of a simultaneous rotation around C5=C6, C6–C7, and C7=C8 bonds, but the directionality of these rotations is different from that in the reaction path leading to S_1_/S_0_-MECI-C6. As the system moves toward S_1_/S_0_-MECI-C7, the energy of the S_1_ state at first rises, then reaches a maximum and falls somewhat. Meanwhile, the energy of the S_0_ state rises rapidly until the system reaches S_1_/S_0_-MECI-C7.

At S_1_/S_0_-MECI-C7, the direction of the reaction path changes. Namely, rotation around the C7=C8 bond continues in the same direction as before, toward a *Z* configuration, but rotation around C5=C6 and C6–C7 bonds reverses direction. While this is happening, the energy of the S_0_ state falls sharply. This demonstrates that S_1_ → S_0_ internal conversion at S_1_/S_0_-MECI-C7 can be followed by isomerization around the C7=C8 bond.

## 4 Conclusions

In this study, the photoisomerization reaction of all-*trans*-RAc was investigated by exploring the ground- and excited-state PESs of two model polyenes: OT and 26DMDP. Three possible mechanisms were considered. The first was the mechanism formulated by Jayathirtha Rao and Bhalerao,^34^ according to which photoisomerization proceeds through a zwitterionic intermediate in which the polyenic chain is twisted around the isomerizing double bond. However, this mechanism is incompatible with the available spectroscopic data. Moreover, our simulations of 26DMDP as a model of RAc provide no evidence for the existence of a zwitterionic species, which could potentially act as an intermediate in the photoisomerization reaction.

The second mechanism that was taken into consideration was the one-bond-flip mechanism proposed by Qu and Liu,^77^ in which isomerization is associated with S_2_ → S_1_ internal conversion. As discussed at more length in Section S2 of the Supporting Information, the question of whether that mechanism is likely to be involved in the photoisomerization of all-*trans*-RAc was addressed by mapping out the S_2_/S_1_ CI seam of *tEtEt*-OT, a representative linear polyene. We determined that the lowest-energy point along the S_2_/S_1_ CI seam is located at a planar molecular geometry, from which we conclude that S_2_ → S_1_ predominantly takes place at planar and near-planar geometries. This provides a strong argument against the involvement of the one-bond-flip mechanism in the case of all-*trans*-RAc.

The most plausible explanation for the photoisomerization reaction of all-*trans*-RAc appears to be the chain-kinking mechanism of Olivucci et al.^51−55^ The operation of that mechanism was investigated by locating the ground- and excited-state minima and CIs of the model compound *tEtEtEc*-26DMDP. We found that *tEtEtEc*-26DMDP exhibits a series of MECI structures between S_1_ and S_0_ states, each of which is associated with a kinking deformation at a different position along the polyenic chain. These S_1_/S_0_-MECIs can act as gateways for *E* → *Z* isomerizations, which lead to mono-*Z* isomers of 26DMDP.

We hypothesize that the photoisomerization reaction of all-*trans*-RAc owes its high degree of regioselectivity to differences in the accessibility of various S_1_/S_0_-MECIs. In this scenario, S_1_/S_0_-MECIs that are the lowest in energy make the greatest contribution to the overall rate of internal conversion to the S_0_ state and are predominantly responsible for the occurrence of photoisomerization. If we take the relative energies calculated at the XMS(2)-CASPT2/cc-pVDZ level at face value, then internal conversion to S_0_ should predominantly take place at S_1_/S_0_-MECI-C7 and S_1_/S_0_-MECI-C6. This would indicate a preference for *E* → *Z* isomerization around the C5=C6 and C7=C8 bonds of *tEtEtEc*-26DMDP, which corresponds to the formation of the 7-*cis* and 9-*cis* isomers of RAc. The prediction of a preference for the formation of these isomers is qualitatively consistent with the actual product distribution of the photoisomerization reaction, in which 9-*cis* isomer is the main product and the 7-*cis* isomer appears as a minor side product.

An important feature of the chain-kinking mechanism is an activated process. As a consequence, the quantum yield and product distribution of the photoisomerization reaction is expected to be sensitive to the irradiation wavelength. We note here that the wavelength dependence of the product distribution was analyzed in ref.^21^ Unfortunately, however, no conclusions regarding the quantum yield of photoisomerization can be drawn, as the reaction yield was not normalized to the molar extinction coefficient of all-*trans*-RAc at different wavelengths and to the energy output of the monochromatic diodes used in those experiments.

Our success in identifying the reaction mechanism notwithstanding, the present study cannot be the final word on the photoisomerization of all-*trans*-RAc, as our calculations are performed on isolated molecules and do not cover solvent effects. This problem will be addressed in a future study, where we hope to include solvent effects via the polarizable continuum model.^120,121^

## Supporting Information Available

Choice of active space in CASSCF calculations; investigation of the role of the one-bond-flip mechanism in the photoisomerization reaction of all-*trans*-RAc; XMS(6)-CASPT2/cc-pVDZ excited-state geometry of *tEtEtEc*-26DMDP; and molecular geometries in Cartesian coordinates (PDF)
