# Supplementary material for: Theoretical Study of the Photoisomerization Mechanism of All-Trans-Retinyl Acetate
Source: J Phys Chem A. 2021 Sep 21;125(38):8358–72. doi: 10.1021/acs.jpca.1c05533 (PMC8488936; doi:10.1021/acs.jpca.1c05533)
Supplement: Supplementary file 1 — jp1c05533_si_001.pdf [file jp1c05533_si_001.pdf]

Supporting Information for

Theoretical Study of the Photoisomerization Mechanism of All-*trans*-Retinyl Acetate

Michał Andrzej Kochman,[*,†] Krzysztof Palczewski,[‡,¶,§] Adam Kubas[*,†]

[†] Institute of Physical Chemistry, Polish Academy of Sciences, Ul. Marcina Kasprzaka 44/52, 01-224 Warszawa, Poland.
[‡] Department of Ophthalmology, Gavin Herbert Eye Institute, University of California, Irvine, California, USA 92697
[¶] Department of Physiology and Biophysics, University of California, Irvine, California, USA 92697
[§] Department of Chemistry, University of California, Irvine, California, USA 92697

e-mail: mkochman@ichf.edu.pl, akubas@ichf.edu.pl

Contents

S1 Choice of Active Space in CASSCF Calculations **S2**

S2 Role of the One-Bond-Flip Mechanism in the Photoisomerization of All-*trans*-RAc **S4**

S2.1 0–0 Excitation Energies of *tEtEt*-1,3,5,7-Octatetraene (*tEtEt*-OT) . . . S4

S2.2 $S_2 \rightarrow S_1$ Internal Conversion . . . S6

S3 XMS(6)-CASPT2 Excited-State Geometry of *tEtEtEc*-26DMDP **S8**

S4 Molecular Geometries **S9**

S4.1 1,3,5,7-Octatetraene (OT) . . . S9

S4.2 2,6-Dimethyl-1,3,5,7,9-decapentaene (26DMDP) . . . S13

S1 Choice of Active Space in CASSCF Calculations

This section shows plots of the active space orbitals employed in the CASSCF calculations for *tEtEt*-OT and *tEtEtEc*-26DMDP. For either molecule, the orbitals were generated through a SA-2-CASSCF/cc-pVDZ calculation at the ground-state equilibrium geometry optimized at the XMS(2)-CASPT2/cc-pVDZ level of theory.

Figure S1: CASSCF active space natural orbitals of *tEtEt*-OT, plotted in the form of isosurfaces with isovalues of $\pm 0.05\ {a_0}^{-3/2}$. Symmetry labels are given in brackets.

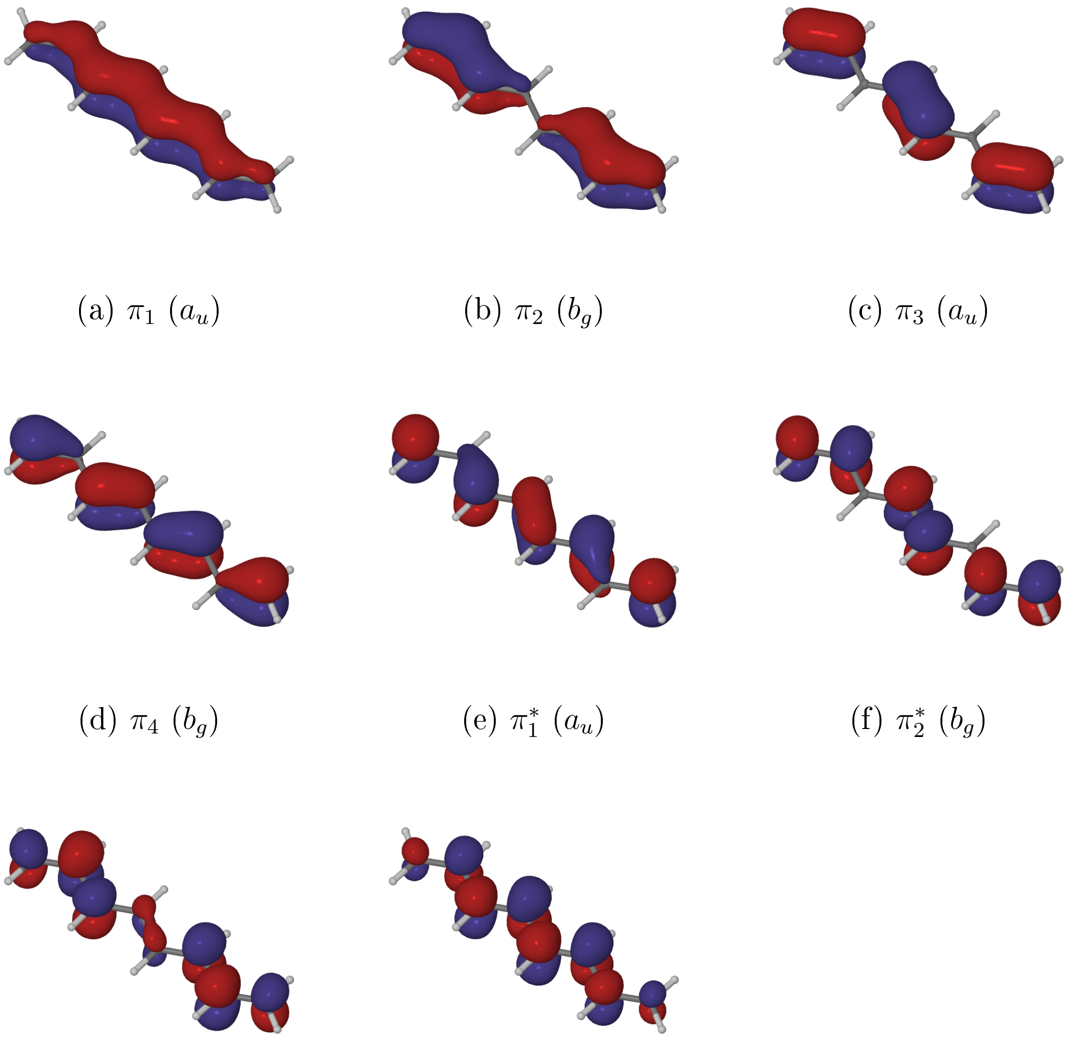

(a) π_1 (a_u) (b) π_2 (b_g) (c) π_3 (a_u)

(d) π_4 (b_g) (e) π_1^* (a_u) (f) π_2^* (b_g)

(g) π_3^* (a_u) (h) π_4^* (b_g)

Figure S2: CASSCF active space natural orbitals of *tEtEtEc*-26DMDP, plotted in the form of isosurfaces with isovalues of $\pm 0.05\ {a_0}^{-3/2}$. The orbitals have been assigned approximate symmetry labels of the C_{2h} point group.

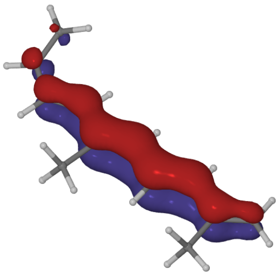

(a) π_1 (a_u-like)

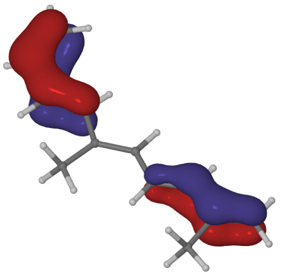

(b) π_2 (b_g-like)

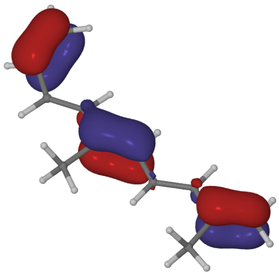

(c) π_3 (a_u-like)

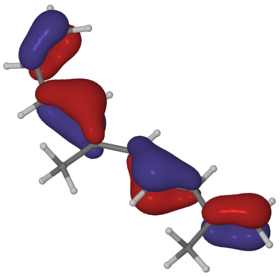

(d) π_4 (b_g-like)

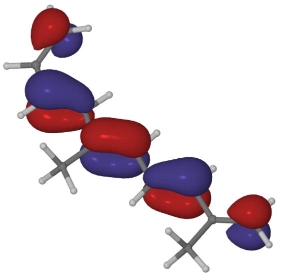

(e) π_5 (a_u-like)

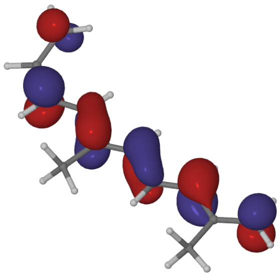

(f) π_1^* (b_g-like)

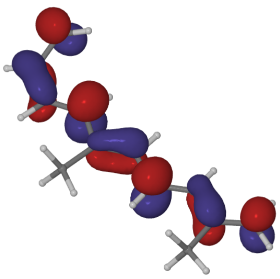

(g) π_2^* (a_u-like)

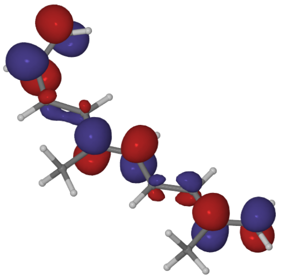

(h) π_3^* (b_g-like)

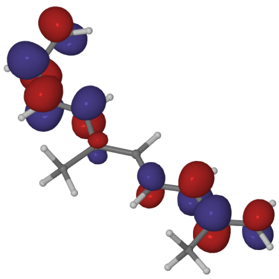

(i) π_4^* (a_u-like)

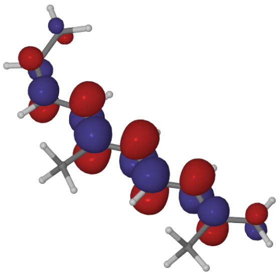

(j) π_5^* (b_g-like)

S2 Role of the One-Bond-Flip Mechanism in the Photoisomerization of All-*trans*-RAc

S2.1 0–0 Excitation Energies of *tEtEt*-1,3,5,7-Octatetraene (*tEtEt*-OT)

tEtEt-1,3,5,7-Octatetraene (*tEtEt*-OT) is our model compound for the purpose of determining whether the photoisomerization reaction of all-*trans*-RAc can take place via the one-bond-flip mechanism proposed by Qu and Liu.[1] As discussed in the Introduction section, this mechanism requires the polyene to undergo S_2 (1 ^1B_u)→S_1 (2 ^1A_g) internal conversion at a substantially twisted geometry. The internal conversion process is mediated by a conical intersection (CI) seam between the S_2 and S_1 states. Therefore, in order to determine where the internal conversion process takes place, we decided to map out the topography of the S_2/S_1 CI seam (which is to say, the set of molecular geometries where the S_2 and S_1 states are degenerate).

Clearly, the characterization of the topography of the CI seam calls for an accurate description of the intersecting states. For this reason, as a preliminary step, we assessed the accuracy of the XMS-CASPT2 method for the excitation energies of octatetraene. In order to take into account the relaxation of molecular geometry in the excited states, we performed the benchmark by calculating the 0–0 transition energies for the S_1 (2 ^1A_g) and the S_2 (1 ^1B_u) states of *tEtEt*-OT, and comparing the results to spectroscopic data. For reasons explained in the Computational Methods section, these calculations were performed with the inclusion of six states in the XMS-CASPT2 calculation, which is denoted as XMS(6)-CASPT2. In order to gain a measure of basis set size effects, the calculations were performed with two basis sets: cc-pVDZ and cc-pVTZ.

The calculated 0–0 transition energies are listed in Table S1. Accompanying this data, Figure S3 (a-c) shows the ground- and excited-state equilibrium geometries of *tEtEt*-OT as optimized at the XMS(6)-CASPT2/cc-pVDZ level.

In agreement with experiment, our calculations predict that that the 0–0 excitation energy into the S_2 (1 ^1B_u) state is higher than that into the S_1 (2 ^1A_g) state. For the S_1 (2 ^1A_g) state, the XMS(6)-CASPT2/cc-pVTZ calculation gives a 0–0 excitation energy of 3.523 eV, which coincides closely with the transition energy (band origin) of 3.589 eV reported by Petek et al.[2] Regarding, in turn, the S_2 (1 ^1B_u) state, the XMS(6)-CASPT2/cc-pVTZ calculation gives a 0–0 excitation energy of 4.122 eV. This is roughly 0.3 eV lower than the value of 4.408 eV determined experimentally by Heimbrook et al.[3,4] It seems that the XMS(6)-CASPT2/cc-pVTZ calculation artificially stabilizes the minimum on the S_2 (1 ^1B_u) state relative to the minimum on the S_1 (2 ^1A_g) state. Nevertheless, the error is small enough that the XMS(6)-CASPT2 method can be expected to provide a realistic picture of the CI seam between these states.

Decreasing the basis set size from cc-pVTZ to cc-pVDZ causes only minor changes in the calculated 0–0 excitation energies, on the order of 0.1 eV. For this reason, we decided to employ the computationally more efficient cc-pVDZ basis set in all further calculations for *tEtEt*-OT.

Table S1: Comparison of calculated 0–0 excitation energies (in eV) of *tEtEt*-OT with experimental data.

Method	S_1 (2 ^1A_g)	S_2 (1 ^1B_u)
XMS(6)-CASPT2/cc-pVDZ	3.470	4.252
XMS(6)-CASPT2/cc-pVTZ	3.523	4.122
Exptl.	3.589 ^{a}	4.408 ^{b}

^{a} Ref.[2]
^{b} Refs.[3,4]

Figure S3: Relevant geometries of *tEtEt*-OT as optimized at the XMS(6)-CASPT2/cc-pVDZ level of theory. Selected bond lengths are given in units of ångström.

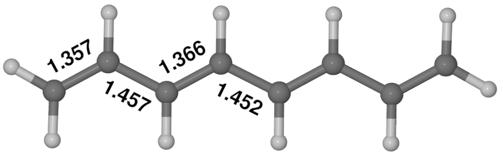

(a) Minimum on the S_0 (1 ^1A_g) state

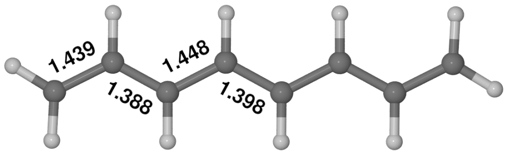

(b) Minimum on the S_1 (2 ^1A_g) state

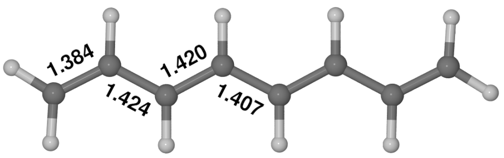

(c) Minimum on the S_2 (1 ^1B_u) state

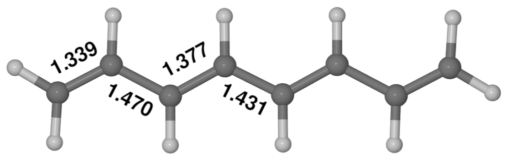

(d) Minimum-energy S_2/S_1 conical intersection geometry (S_2/S_1-MECI)

S2.2 $S_2 \rightarrow S_1$ Internal Conversion

We now return to the problem of where the S_2 ($1\,^1B_u$)$\rightarrow$$S_1$ ($2\,^1A_g$) internal conversion takes place. Because the internal conversion process can be assumed to take place in the vicinity of the S_2/S_1 CI seam, the molecular geometries where it occurs can be determined by mapping out the topography of that seam in the space spanned by the internal degrees of freedom of the molecule. Presently, we are interested in whether the internal conversion occurs at planar or at twisted molecular geometries. Therefore, we scanned the energy of the S_2/S_1 CI seam as a function of the torsion angle of one of the inner C=C bonds of *tEtEt*-OT. More specifically, the scan was performed with the two geometric constraints shown in Figure S4 (a). Throughout the scan, the C4-C5-C6-C7 torsion angle (denoted τ_{4567}) was always constrained at 180°. The C2-C3-C4-C5 torsion angle (τ_{2345}) was constrained at values ranging from 140° to 220° in steps of 5°. For each value of τ_{2345} (or, each scan point), the penalty function was minimized in the remaining internal degrees of freedom. This procedure amounts to a scan of the energy of the S_2/S_1 CI seam as a function of τ_{2345}.

The reason that τ_{4567} was constrained during the scan is that the C3=C4 and C5=C6 bonds are equivalent by symmetry. If τ_{2345} is to be varied, then τ_{4567} must be constrained, or else the scan would be meaningless. Because only two degrees of freedom (τ_{2345} and τ_{4567}) were constrained during the scan along the S_2/S_1 CI seam, and all other degrees of freedom were re-optimized at each scan point, it was effectively a relaxed scan.

The results of the scan are presented in Figure S4 (b). Because of the symmetry of the *tEtEt*-OT molecule, the energy of the S_2/S_1 CI seam is even with respect to the point $\tau_{2345} = 180°$. The point $\tau_{2345} = 180°$ also corresponds to the minimum-energy point along the S_2/S_1 CI seam (S_2/S_1-MECI). It is located 4.606 eV higher in energy than the S_0 (^1A_g) state at the ground-state equilibrium geometry. The S_2/S_1-MECI geometry, shown in Figure S3 (d), is planar and exhibits C_{2h} symmetry.

An analogy can be drawn to the case of the shorter polyene *tEt*-1,3,5-hexatriene (*tEt*-HT). According to Garavelli et al.,[5] in *tEt*-HT, motion from the minimum on the S_2 ($1\,^1B_u$) state to the S_2/S_1 CI seam proceeds along a totally symmetric pathway. Our results indicate that the situation is the same in *tEtEt*-OT: the minimum on the S_2 ($1\,^1B_u$) state and the S_2/S_1 MECI structure both exhibit ideal C_{2h} symmetry. It follows that in *tEtEt*-OT, motion from the S_2 ($1\,^1B_u$) minimum to the S_2/S_1 MECI also takes place along a totally symmetric path.

As the OT molecule twists around the C5=C6 bond, and τ_{2345} deviates from 180°, the energy of the CI seam rises. Also, it can be seen in Figure S4 (b) that the slope of the CI energy curve suddenly changes at $\tau_{2345} \approx 140°$ and likewise at $\tau_{2345} \approx 220°$. This is due to an artifact in the reference CASSCF calculation: the diabatic character of one of the states included in the state-averaging scheme changes suddenly on going from the $\tau_{2345} = 145°$ point to the $\tau_{2345} = 140°$ point. The same happens on going from the $\tau_{2345} = 215°$ point to the $\tau_{2345} = 220°$ point. This discontinuity in the CASSCF wavefunction means that the scan cannot be extended to τ_{2345} values below 140°, or above 220°.

Although the scan only covers a limited range of τ_{2345}, some conclusions can be

drawn regarding the likelihood of S_2 ($1\,^1B_u$)$\rightarrow$$S_1$ ($2\,^1A_g$) internal conversion taking place at different segments of the S_2/S_1 CI seam. At the XMS(6)-CASPT2/cc-pVDZ level of theory, the vertical excitation energy into the bright S_2 ($1\,^1B_u$) state is 4.580 eV. This is slightly lower than the energy of the S_2/S_1-MECI, which is 4.606 eV. Thus, when *tEtEt*-OT is excited near the band origin of the S_2 ($1\,^1B_u$) state, it can only reach the lowermost segment of the S_2/S_1 CI seam, which corresponds to planar and near-planar geometries. At higher excitation energies, the molecule can access the S_2/S_1 CI seam over a progressively broader range of torsion angles. Even so, internal conversion will predominantly take place at planar and near-planar geometries, because the initial geometry upon photoexcitation (i.e. the Franck-Condon geometry, which is the ground-state equilibrium geometry) and the MECI geometry are both planar.

The preference for internal conversion at planar and near-planar geometries has implications for the photoisomerization mechanism of all-*trans*-RAc. As discussed in Section 3.1 in the main body of our paper, the S_1 and S_2 excited states of all-*trans*-RAc are similar in terms of structure to those of *tEtEt*-OT and other linear polyenes, so the topography of the S_2/S_1 CI seam is likewise expected to be similar. In the synthesis developed by Kahremany et al.,[6] a solution of all-*trans*-RAc is irradiated at 385 nm, close to the origin of the first photoabsorption band. By analogy to the case of *tEtEt*-OT, this favors internal conversion taking place at near-planar geometries. There is also another factor that will promote internal

Figure S4: (a) Schematic illustration of the PES scan along the S_2/S_1 CI seam of OT. The C4-C5-C6-C7 torsion angle (τ_{4567}) was constrained at 180°, and the C2-C3-C4-C5 torsion angle (τ_{2345}) was varied in the range from 140° to 220° in increments of 5°. The remaining internal coordinates were re-optimized at each scan point (a so-called relaxed scan).
(b) Energy of the S_2/S_1 CI seam of OT as a function of τ_{2345}. The zero of the energy scale corresponds to the energy of the S_0-min (*tEtEt*) structure as optimized at the XMS(6)-CASPT2 level.

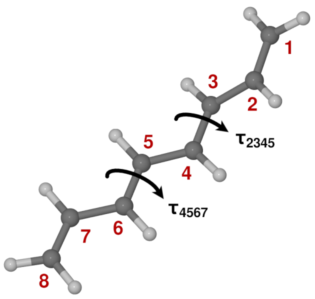

(a)

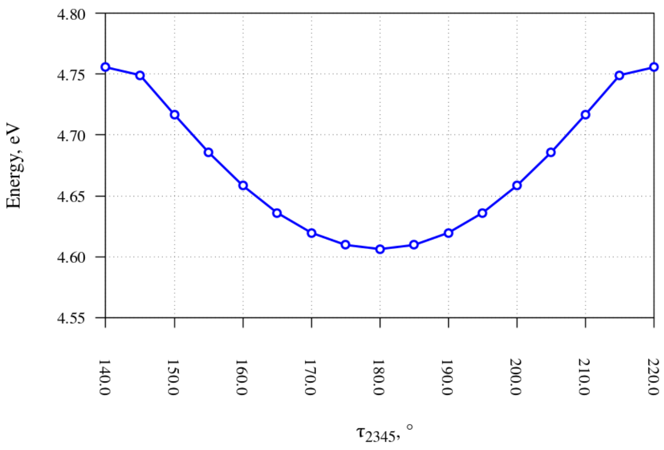

(b)

conversion at near-planar geometries. The polyenic chain of RAc has a β-ionone ring on one end, and an ester group on the other. Both these moieties are quite heavy, and give rise to a large moment of inertia along the torsional coordinate. This means that internal conversion at near-planar geometries is also favored on kinetic grounds – it will occur before the polyenic chain has had the chance to undergo a significant twist.

In summary, we expect that the $S_2 \rightarrow S_1$ process of all-*trans*-RAc will overwhelmingly take place at near-planar geometries of the polyenic chain. It follows that its photoisomerization reaction cannot proceed through the one-bond-flip mechanism predicted by Qu and Liu[1] for *tEtEt*-OT. By default, this leaves the chain-kinking mechanism as the most plausible scenario.

S3 XMS(6)-CASPT2 Excited-State Geometry of *tEtEtEc*-26DMDP

In Figure S5, we show the geometry of the minimum on the PES of the S_1 state of *tEtEtEc*-26DMDP, as optimized at the XMS(6)-CASPT2/cc-pVDZ level of theory. This geometry is almost identical to that obtained in the two-state calculation (XMS(2)-CASPT2/cc-pVDZ; see Figure 6 (b) in the main body of our paper). This observation confirms that mixing between the $2\ ^1A_g$ and $1\ ^1B_u$ states does not come into play at the excited-state minimum, and that the S_1 state at this geometry has nearly pure $2\ ^1A_g$ character.

Figure S5: Equilibrium geometry of *tEtEtEc*-26DMDP on the PES of the S_1 state (S_1-min), as optimized at the XMS(6)-CASPT2/cc-pVDZ level of theory.

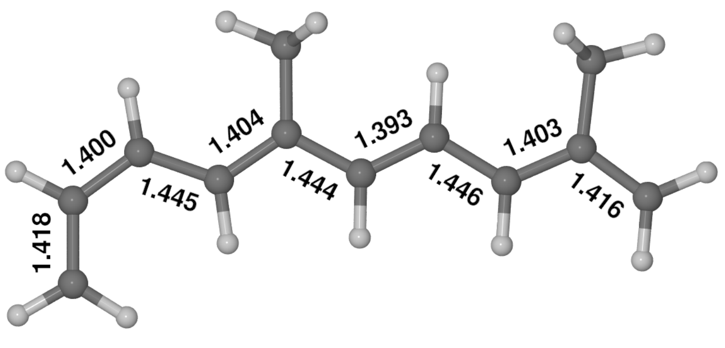

S4 Molecular Geometries

In order to ensure that our results can be reproduced by other researchers, the present section lists selected geometries of OT and 26DMDP. All geometries are given in terms of Cartesian coordinates in units of ångström (Å).

S4.1 1,3,5,7-Octatetraene (OT)

Ground-state equilibrium geometry of *tEtEt*-OT, optimized at the XMS(6)-CASPT2/cc-pVDZ level of theory:

```
C       1.135414    2.942337    0.000000
C       2.263301    3.697508    0.000000
C       1.136600    1.484916    0.000000
C       0.001134    0.726118    0.000000
C      -0.001134   -0.726118    0.000000
C      -1.136600   -1.484916    0.000000
C      -1.135414   -2.942337    0.000000
C      -2.263301   -3.697508    0.000000
H       0.156060    3.434804    0.000000
H       3.253819    3.233315    0.000000
H       2.218593    4.788467    0.000000
H       2.113029    0.984479    0.000000
H      -0.974416    1.228332    0.000000
H       0.974416   -1.228332    0.000000
H      -2.113029   -0.984479    0.000000
H      -0.156060   -3.434804    0.000000
H      -3.253819   -3.233315    0.000000
H      -2.218593   -4.788467    0.000000
```

Minimum on the PES of the S_1 ($2\ ^1A_g$) state of *tEtEt*-OT, optimized at the XMS(6)-CASPT2/cc-pVDZ level of theory:

C	1.143694	2.919119	-0.000394
C	2.317475	3.751054	-0.000537
C	1.170813	1.531089	0.000071
C	-0.014616	0.698803	0.000204
C	0.014625	-0.698809	0.000559
C	-1.170802	-1.531093	0.000691
C	-1.143691	-2.919122	0.001176
C	-2.317489	-3.751048	0.001322
H	0.166307	3.415409	-0.000667
H	3.313750	3.303945	-0.000317
H	2.235869	4.837950	-0.000831
H	2.143627	1.024022	0.000345
H	-0.988342	1.203297	0.000004
H	0.988351	-1.203301	0.000761
H	-2.143616	-1.024025	0.000403
H	-0.166311	-3.415424	0.001459
H	-3.313755	-3.303922	0.001175
H	-2.235889	-4.837944	0.001577

Minimum on the PES of the S_2 ($1\ ^1B_u$) state of *tEtEt*-OT, optimized at the XMS(6)-CASPT2/cc-pVDZ level of theory:

C	1.154454	2.925641	-0.000386
C	2.283869	3.725147	-0.000525
C	1.173065	1.501917	0.000070
C	-0.001640	0.703553	0.000200
C	0.001640	-0.703553	0.000573
C	-1.173065	-1.501917	0.000701
C	-1.154454	-2.925641	0.001171
C	-2.283870	-3.725146	0.001306
H	0.167843	3.408556	-0.000641
H	3.286838	3.284283	-0.000283
H	2.205517	4.815879	-0.000877
H	2.145481	0.990771	0.000328
H	-0.971393	1.219312	-0.000012
H	0.971393	-1.219313	0.000789
H	-2.145480	-0.990771	0.000432
H	-0.167843	-3.408557	0.001438
H	-3.286838	-3.284283	0.001049
H	-2.205517	-4.815878	0.001668

S_2/S_1-MECI of *tEtEt*-OT, optimized at the XMS(6)-CASPT2/cc-pVDZ level of theory:

C	1.155451	2.943298	0.000000
C	2.257329	3.703937	0.000000
C	1.160969	1.473165	0.000000
C	0.011475	0.715309	0.000000
C	-0.011478	-0.715306	0.000000
C	-1.160969	-1.473164	0.000000
C	-1.155449	-2.943297	0.000000
C	-2.257328	-3.703940	0.000000
H	0.162125	3.416824	0.000000
H	3.261911	3.258359	0.000000
H	2.192769	4.799321	0.000000
H	2.135320	0.960425	0.000000
H	-0.957806	1.236260	0.000000
H	0.957804	-1.236256	0.000000
H	-2.135322	-0.960427	0.000000
H	-0.162122	-3.416821	0.000000
H	-3.261911	-3.258363	0.000000
H	-2.192768	-4.799324	0.000000

S4.2 2,6-Dimethyl-1,3,5,7,9-decapentaene (26DMDP)

Ground-state equilibrium geometry of *tEtEtEc*-26DMDP (S_0-min (*tEtEtEc*)), optimized at the XMS(2)-CASPT2/cc-pVDZ level of theory:

C	2.614517	-1.129737	-0.269313
C	1.652883	-0.199263	-0.016788
C	0.240819	-0.557099	-0.158978
C	-0.814324	0.284335	0.065005
C	-2.193877	-0.137111	-0.095013
C	-3.325291	0.617879	0.100700
C	-4.623568	-0.015842	-0.124146
C	-5.839211	0.586141	0.035906
H	3.676501	-0.892589	-0.171945
H	2.350037	-2.144684	-0.577897
C	2.010091	1.203886	0.409723
H	0.030337	-1.587626	-0.470898
H	-0.615174	1.312676	0.376314
H	-2.336848	-1.178749	-0.409300
H	-4.597034	-1.067356	-0.433643
H	-5.882570	1.615110	0.406701
C	-3.304179	2.062569	0.533103
H	3.100246	1.328732	0.474168
H	1.576461	1.438633	1.395414
H	1.619045	1.945038	-0.306171
H	-2.285237	2.447526	0.662834
H	-3.837775	2.187025	1.489744
H	-3.815261	2.695586	-0.210467
C	-7.130269	-0.066564	-0.222975
C	-7.337081	-1.076625	-1.105946
H	-7.989376	0.323989	0.333981
H	-6.529012	-1.465650	-1.731047
H	-8.326121	-1.523707	-1.226390

Excited-state equilibrium geometry of *tEtEtEc*-26DMDP (S_1-min (*tEtEtEc*)), optimized at the XMS(2)-CASPT2/cc-pVDZ level of theory:

C	2.638095	-1.121585	-0.288494
C	1.599662	-0.204636	-0.002303
C	0.258944	-0.566053	-0.212918
C	-0.883029	0.280720	0.041135
C	-2.193737	-0.134503	-0.188006
C	-3.380352	0.653379	0.041443
C	-4.628563	0.076141	-0.245022
C	-5.926064	0.689623	-0.087237
H	3.685119	-0.857805	-0.130947
H	2.410525	-2.117687	-0.675159
C	1.947175	1.168064	0.532439
H	0.057171	-1.571340	-0.601585
H	-0.703161	1.286796	0.428875
H	-2.345355	-1.148980	-0.577320
H	-4.605281	-0.948725	-0.628461
H	-5.977154	1.711947	0.293703
C	-3.219701	2.053517	0.576040
H	3.035099	1.285386	0.637316
H	1.490386	1.338033	1.520881
H	1.583270	1.961599	-0.140197
H	-2.607840	2.662648	-0.110233
H	-2.700605	2.039751	1.549043
H	-4.179862	2.565231	0.714458
C	-7.141266	0.061956	-0.390795
C	-7.307221	-1.252468	-0.893525
H	-8.051225	0.646823	-0.222090
H	-6.460251	-1.909965	-1.093017
H	-8.306048	-1.641348	-1.095346

S_1/S_0-MECI-C2 of *tEtEtEc*-26DMDP, optimized at the XMS(2)-CASPT2/cc-pVDZ level of theory:

C	2.056763	-1.246851	0.298725
C	1.694056	-0.013933	-0.432834
C	0.351915	-0.421337	-0.546109
C	-0.813517	0.335061	-0.076230
C	-2.096876	-0.059798	-0.342837
C	-3.307689	0.647895	0.056509
C	-4.535204	0.064713	-0.244119
C	-5.824488	0.576196	0.069136
H	1.943527	-1.274057	1.390449
H	2.207798	-2.200828	-0.215396
C	2.124299	1.349283	0.013078
H	0.135932	-1.368660	-1.054780
H	-0.624529	1.260582	0.475103
H	-2.241941	-0.996705	-0.895683
H	-4.493076	-0.889644	-0.780198
H	-5.894326	1.518775	0.616672
C	-3.157135	1.957505	0.784856
H	3.214419	1.464010	-0.094584
H	1.884779	1.530943	1.080722
H	1.630478	2.134644	-0.581318
H	-2.555289	2.661992	0.187682
H	-2.630384	1.809577	1.742363
H	-4.121450	2.434173	0.998656
C	-7.060310	-0.053011	-0.276015
C	-7.225479	-1.236777	-0.966482
H	-7.967295	0.469147	0.046574
H	-6.380484	-1.824857	-1.329955
H	-8.225767	-1.621521	-1.171303

S_1/S_0-MECI-C3 of *tEtEtEc*-26DMDP, optimized at the XMS(2)-CASPT2/cc-pVDZ level of theory:

C	-2.943369	-0.068589	0.774203
C	-1.597181	0.358457	0.435865
C	-1.173144	0.032831	-0.883202
C	-0.057652	-0.859565	-0.546563
C	-0.078385	-2.247047	-0.766930
C	0.971035	-3.151221	-0.493008
C	0.846555	-4.494743	-0.965199
C	1.808724	-5.486034	-0.846990
H	-3.749660	0.674508	0.826759
H	-3.227808	-1.109155	0.599823
C	-0.762453	1.120142	1.424800
H	-1.932866	-0.327136	-1.588891
H	0.790381	-0.421502	-0.013246
H	-0.958706	-2.654381	-1.279807
H	-0.093174	-4.739044	-1.473287
H	2.766515	-5.231972	-0.384394
C	2.226265	-2.719814	0.223990
H	-1.344445	2.012127	1.719903
H	-0.584489	0.539603	2.343765
H	0.190627	1.457985	0.993088
H	2.073752	-1.792215	0.791849
H	2.550718	-3.492747	0.936904
H	3.054737	-2.553244	-0.485405
C	1.670440	-6.857583	-1.312516
C	0.545566	-7.440798	-1.820265
H	2.570247	-7.476833	-1.231854
H	-0.399768	-6.901344	-1.914455
H	0.559546	-8.482355	-2.146387

S_1/S_0-MECI-C4 of *tEtEtEc*-26DMDP, optimized at the XMS(2)-CASPT2/cc-pVDZ level of theory:

C	1.320997	-2.480476	-0.045443
C	0.213206	-1.895298	-0.619930
C	0.048858	-0.467774	-0.561540
C	-1.010819	0.312819	-1.247555
C	-1.757888	0.275673	-0.066343
C	-3.176103	-0.143730	0.107715
C	-3.720515	-0.041118	1.367581
C	-5.090614	-0.294615	1.734314
H	1.505021	-3.553504	-0.132709
H	2.056402	-1.881001	0.498150
C	-0.794189	-2.734815	-1.371493
H	0.768943	0.079050	0.062779
H	-1.444823	-0.024287	-2.192508
H	-1.299828	0.684805	0.843816
C	-3.946894	-0.502269	-1.132877
H	-3.034756	0.249091	2.171255
H	-5.820371	-0.451171	0.936933
H	-1.798619	-2.617035	-0.933104
H	-0.524618	-3.799768	-1.324538
H	-0.857126	-2.444025	-2.432138
H	-3.404560	-1.258544	-1.721129
H	-4.945876	-0.897329	-0.908162
H	-4.061173	0.384345	-1.780009
C	-5.568584	-0.350739	3.066628
C	-4.813826	-0.220550	4.225149
H	-6.644342	-0.517068	3.186137
H	-5.292533	-0.270049	5.204202
H	-3.732327	-0.075398	4.203362

S_1/S_0-MECI-C5 of *tEtEtEc*-26DMDP, optimized at the XMS(2)-CASPT2/cc-pVDZ level of theory:

C	1.341348	-2.782947	-0.531650
C	0.196888	-1.986787	-0.644088
C	0.176415	-0.584231	-0.436739
C	-0.997255	0.205072	-0.875536
C	-1.559855	0.161154	0.414590
C	-2.904523	-0.348435	0.684922
C	-3.822144	0.300350	1.546025
C	-5.131279	-0.160379	1.656502
H	2.262171	-2.316121	-0.169533
H	-0.729229	-2.434657	-1.019780
C	1.335650	0.141004	0.194261
H	-1.606734	-0.172960	-1.706232
H	-0.968429	0.537130	1.257924
H	-3.263898	-1.177124	0.066209
C	-3.360596	1.485842	2.367460
H	-5.478616	-1.004674	1.056013
H	-5.835243	0.293324	2.356836
H	-3.003710	2.300869	1.718257
H	-4.177768	1.874837	2.991249
H	-2.528097	1.205215	3.033074
H	1.012767	1.118919	0.579693
H	2.087938	0.339641	-0.589388
H	1.816715	-0.432804	1.000196
C	1.427754	-4.183497	-0.876771
C	0.436612	-4.958594	-1.419978
H	2.392739	-4.662274	-0.678267
H	-0.549075	-4.556874	-1.665753
H	0.611074	-6.015431	-1.629775

S_1/S_0-MECI-C6 of *tEtEtEc*-26DMDP, optimized at the XMS(2)-CASPT2/cc-pVDZ level of theory:

C	-3.006885	-0.247196	1.016312
C	-1.606025	0.036030	0.696821
C	-1.155614	0.081887	-0.641698
C	-0.131729	-0.942295	-0.379139
C	-0.205676	-2.291746	-0.805204
C	0.820574	-3.207772	-0.582144
C	0.861716	-4.559184	-1.096569
C	1.929650	-5.370057	-0.812453
H	-3.480569	-1.094990	0.512749
H	-0.891533	0.229473	1.508051
C	-2.075083	0.030291	-1.826777
H	0.729245	-0.655205	0.240293
H	-1.071561	-2.594791	-1.401696
H	1.679619	-2.881105	0.017251
C	-0.287746	-5.059918	-1.940371
H	2.762765	-5.010277	-0.202890
H	1.979671	-6.396163	-1.183305
H	-2.850927	0.807087	-1.735542
H	-2.595591	-0.940081	-1.943268
H	-1.504834	0.203729	-2.753993
H	-1.237022	-5.015592	-1.382067
H	-0.121432	-6.101110	-2.251325
H	-0.410352	-4.445104	-2.846787
C	-3.794170	0.623333	1.795075
C	-3.335107	1.801823	2.376570
H	-4.842393	0.345243	1.950957
H	-2.313092	2.152628	2.215877
H	-3.993679	2.417382	2.991280

S_1/S_0-MECI-C7 of *tEtEtEc*-26DMDP, optimized at the XMS(2)-CASPT2/cc-pVDZ level of theory:

C	2.443030	-0.657810	0.231917
C	1.389929	-0.143177	-0.650712
C	0.372524	-1.063641	-0.287396
C	-0.806388	-0.497841	0.370950
C	-2.098655	-0.573320	-0.162132
C	-3.210995	0.086770	0.387046
C	-4.561046	-0.013586	-0.104319
C	-5.573789	0.666566	0.528626
H	2.831966	-1.662571	0.043436
H	1.144408	0.924027	-0.617075
C	0.431758	-2.540971	-0.562875
H	-0.618969	0.140816	1.243438
H	-2.246771	-1.184406	-1.059389
H	-3.045627	0.715526	1.270115
C	-4.849454	-0.866939	-1.318825
H	-5.371785	1.284292	1.407442
H	-6.605021	0.610898	0.173713
H	-4.278935	-0.516497	-2.193985
H	-4.568961	-1.917646	-1.140921
H	-5.918397	-0.836730	-1.572925
H	1.334679	-2.821351	-1.123327
H	0.355916	-3.134272	0.362000
H	-0.446994	-2.799807	-1.180963
C	2.882918	0.000362	1.422489
C	2.456603	1.234948	1.868177
H	3.643488	-0.525135	2.011141
H	1.694716	1.808785	1.336260
H	2.860953	1.663641	2.787192

S_1/S_0-MECI-C8 of *tEtEtEc*-26DMDP, optimized at the XMS(2)-CASPT2/cc-pVDZ level of theory:

C	-0.560174	0.139433	0.384354
C	0.316416	-0.728543	-0.356913
C	1.662915	-0.389043	-0.839796
C	2.213070	-0.885160	0.382033
C	-0.066561	1.459946	0.917843
H	1.859179	0.677495	-1.004494
H	2.880263	-0.238110	0.977911
H	-0.143820	-1.686911	-0.635300
C	1.824121	-2.100803	0.999565
C	-1.906175	-0.216644	0.387945
H	2.133051	-2.321115	2.023617
H	1.312375	-2.888685	0.444975
H	-2.165102	-1.208963	-0.002185
C	-2.996896	0.607207	0.823974
C	-4.302250	0.167306	0.805619
C	-5.457361	0.941826	1.239505
C	-6.701800	0.386221	1.171752
C	-5.248056	2.345137	1.755396
H	0.987159	1.378782	1.217363
H	-0.137746	2.255941	0.158167
H	-0.651326	1.771394	1.795402
H	-2.776303	1.617792	1.176795
H	-4.501933	-0.847091	0.439415
H	-6.845407	-0.630443	0.796995
H	-7.589473	0.938069	1.488501
H	-4.779331	2.981711	0.987643
H	-4.584701	2.346977	2.635365
H	-6.204545	2.802623	2.044483

S_1/S_0-MECI-C9 of *tEtEtEc*-26DMDP, optimized at the XMS(2)-CASPT2/cc-pVDZ level of theory:

C	2.529414	-1.117581	-0.306881
C	1.525336	-0.211783	-0.042780
C	0.151404	-0.593089	-0.208582
C	-0.960315	0.244763	0.029659
C	-2.280514	-0.167057	-0.149469
C	-3.457094	0.646115	0.087776
C	-4.697454	0.072604	-0.105931
C	-5.996462	0.687436	0.170863
H	3.581655	-0.851155	-0.188040
H	2.296488	-2.130982	-0.643581
C	1.859867	1.187887	0.422984
H	-0.045674	-1.617032	-0.547907
H	-0.771464	1.266279	0.371639
H	-2.453989	-1.195268	-0.489868
H	-4.724241	-0.965239	-0.454697
H	-5.979024	1.631414	0.736517
C	-3.259000	2.072985	0.541324
H	2.947750	1.325139	0.500156
H	1.417652	1.393284	1.411185
H	1.467997	1.943173	-0.277204
H	-2.621499	2.616681	-0.174426
H	-2.754321	2.106500	1.520829
H	-4.204499	2.623032	0.624707
C	-7.239522	0.128054	-0.193561
C	-6.922014	0.125929	-1.630915
H	-8.158202	0.599291	0.166691
H	-6.300373	-0.642257	-2.096176
H	-7.183822	0.994716	-2.245762

Ground-state equilibrium geometry of *tZtEtEc*-26DMDP (S_0-min (*tZtEtEc*)), optimized at the XMS(2)-CASPT2/cc-pVDZ level of theory:

C	2.370989	-0.628776	0.794234
C	1.175676	-0.031313	1.077690
C	-0.132733	-0.470142	0.593491
C	-1.236271	0.248414	0.993974
C	-2.614588	-0.021790	0.622994
C	-3.760213	0.645341	0.980309
C	-3.985692	1.820830	1.830794
C	-5.269389	2.250746	1.994483
H	2.383882	-1.543586	0.193483
H	1.173560	0.846521	1.734852
C	-0.180926	-1.676869	-0.309538
H	-1.039612	1.097001	1.649829
H	-2.774245	-0.879423	-0.036491
H	-4.686165	0.235364	0.560530
C	-2.856324	2.552925	2.516270
H	-5.502834	3.119322	2.614736
H	-6.104752	1.738799	1.509608
H	-2.134827	2.948524	1.784446
H	-2.308108	1.888562	3.202313
H	-3.248630	3.398028	3.100056
H	0.431601	-1.507261	-1.209944
H	0.233881	-2.558800	0.205656
H	-1.198415	-1.924471	-0.634341
C	3.673983	-0.148110	1.274746
C	3.972767	1.140817	1.578250
H	4.463650	-0.902366	1.365742
H	3.242223	1.943842	1.449649
H	4.962190	1.415551	1.949910

Ground-state equilibrium geometry of *tEtZtEc*-26DMDP (S_0-min (*tEtZtEc*)), optimized at the XMS(2)-CASPT2/cc-pVDZ level of theory:

C	-3.282677	0.757355	-1.730116
C	-2.320775	-0.176145	-1.465744
C	-1.146507	0.050193	-0.624912
C	-0.203563	-0.925791	-0.405682
C	-0.193470	-2.278040	-0.938396
C	0.800620	-3.171294	-0.649105
C	0.874844	-4.544852	-1.150011
C	1.919235	-5.340515	-0.789201
H	-3.169763	1.772625	-1.337364
H	-2.429508	-1.161400	-1.927503
C	-0.979953	1.405535	0.018900
H	0.642847	-0.671076	0.243827
H	-1.009693	-2.589830	-1.594499
H	1.616936	-2.849835	0.009657
C	-0.217928	-5.047901	-2.061685
H	2.708058	-4.970722	-0.128672
H	2.001834	-6.369480	-1.146909
H	-0.914873	2.200894	-0.742153
H	-1.838068	1.644831	0.668429
H	-0.066013	1.440164	0.628796
H	-1.199984	-4.999799	-1.563618
H	-0.032621	-6.089993	-2.358219
H	-0.282517	-4.434767	-2.975264
C	-4.473818	0.506743	-2.554064
C	-5.082646	-0.696955	-2.704825
H	-4.905333	1.378027	-3.059456
H	-4.731269	-1.586313	-2.174880
H	-5.954867	-0.808807	-3.352383

Ground-state equilibrium geometry of *tEtEtZc*-26DMDP (S_0-min (*tEtEtZc*)), optimized at the XMS(2)-CASPT2/cc-pVDZ level of theory:

C	2.464229	-0.988096	0.036704
C	1.282362	-0.307669	-0.056135
C	-0.054547	-0.787562	-0.438157
C	-1.128420	-0.016249	-0.064810
C	-2.525502	-0.306984	-0.332641
C	-3.541835	0.510019	0.079294
C	-4.967487	0.268584	-0.151244
C	-5.886947	1.159894	0.310800
H	3.360727	-0.384331	0.224703
H	1.327416	0.765885	0.164768
C	-0.230242	-2.030007	-1.285028
H	-0.928841	0.899775	0.505386
H	-2.765697	-1.226184	-0.873537
H	-3.287285	1.424213	0.629861
C	-5.383979	-0.972542	-0.902323
H	-5.579091	2.057105	0.854515
H	-6.957352	1.007760	0.153571
H	-4.942476	-0.988770	-1.912032
H	-5.043296	-1.881680	-0.380670
H	-6.477441	-1.021720	-1.002943
H	0.688800	-2.255038	-1.841925
H	-0.473638	-2.919484	-0.682586
H	-1.042102	-1.881600	-2.012555
C	2.690174	-2.442334	-0.020171
C	1.935232	-3.355186	0.638831
H	3.581482	-2.783738	-0.560579
H	1.081340	-3.045379	1.247108
H	2.168359	-4.421856	0.593526

References

[1] Qu, Z.; Liu, C. A Non-Adiabatic Dynamics Study of Octatetraene: The Radiationless Conversion from S_2 to S_1. *J. Chem. Phys.* **2013**, *139*, 244304. DOI: 10.1063/1.4853715

[2] Petek, H.; Bell, A. J.; Choi, Y. S.; Yoshihara, K.; Tounge, B. A.; Christensen, R. L. One- and Two-Photon Fluorescence Excitation Spectra of the 2 ^1A_g States of Linear Tetraenes in Free Jet Expansions. *J. Chem. Phys.* **1995**, *102*, 4726-4739. DOI: 10.1063/1.469521

[3] Heimbrook, L. A.; Kenny, J. E.; Kohler, B. E.; Scott, G. W. Free-jet Fluorescence Excitation Spectrum of *trans,trans*-1,3,5,7-Octatetraene. *J. Chem. Phys.* **1981**, *75*, 4338-4342. DOI: 10.1063/1.442640

[4] Heimbrook, L. A.; Kohler, B. E.; Levy, I. J. Fluorescence from the 1 ^1B_u State of *trans,trans*-1,3,5,7-Octatetraene in a Free Jet. *J. Chem. Phys.* **1984**, *81*, 1592-1597. DOI: 10.1063/1.447888

[5] Garavelli, M.; Celani, P.; Bernardi, F.; Robb, M. A.; Olivucci, M. Force Fields for "Ultrafast" Photochemistry: The S_2 ($1B_u$) $\rightarrow$ S_1 ($2A_g$) $\rightarrow$ S_0 ($1A_g$) Reaction Path for *all-trans*-Hexa-1,3,5-triene. *J. Am. Chem. Soc.* **1997**, *119*, 11487-11494. DOI: 10.1021/ja971280u

[6] Kahremany, S.; Sander, C. L.; Tochtrop, G. P.; Kubas, A.; Palczewski, K. Z-isomerization of Retinoids through Combination of Monochromatic Photoisomerization and Metal Catalysis. *Org. Biomol. Chem.* **2019**, *17*, 8125-8139. DOI: 10.1039/c9ob01645g
